# The effect of smoking on DNA methylation of peripheral blood mononuclear cells from African American women

**DOI:** 10.1186/1471-2164-15-151

**Published:** 2014-02-22

**Authors:** Meeshanthini V Dogan, Bridget Shields, Carolyn Cutrona, Long Gao, Frederick X Gibbons, Ronald Simons, Martha Monick, Gene H Brody, Kai Tan, Steven RH Beach, Robert A Philibert

**Affiliations:** 1 Department of Psychiatry, University of Iowa, Rm 2-126 MEB, 500 Newton Road, Iowa City, IA 52242, USA; 2 Department of Biomedical Engineering, University of Iowa, Iowa City, IA, USA; 3 Department of Psychology, Iowa State University, Ames, IA, USA; 4 Department of Psychology, University of Connecticut, Storrs, CT, USA; 5 Department of Sociology, University of Georgia, Athens, GA, USA; 6 Department of Internal Medicine, University of Iowa, Iowa City, IA, USA; 7 Center for Family Research, University of Georgia, Athens, GA, USA

## Abstract

**Background:**

Regular smoking is associated with a wide variety of syndromes with prominent inflammatory components such as cancer, obesity and type 2 diabetes. Heavy regular smoking is also associated with changes in the DNA methylation of peripheral mononuclear cells. However, in younger smokers, inflammatory epigenetic findings are largely absent which suggests the inflammatory response(s) to smoking may be dose dependent. To help understand whether peripheral mononuclear cells have a role in mediating these responses in older smokers with higher cumulative smoke exposure, we examined genome-wide DNA methylation in a group of well characterized adult African American subjects informative for smoking, as well as serum C-reactive protein (CRP) and interleukin-6 receptor (IL6R) levels. In addition, complementary bioinformatic analyses were conducted to delineate possible pathways affected by long-term smoking.

**Results:**

Genome-wide DNA methylation analysis with respect to smoking status yielded 910 significant loci after Benjamini-Hochberg correction. In particular, two loci from the *AHRR* gene (cg05575921 and cg23576855) and one locus from the *GPR15* gene (cg19859270) were identified as highly significantly differentially methylated between smokers and non-smokers. The bioinformatic analyses showed that long-term chronic smoking is associated with altered promoter DNA methylation of genes coding for proteins mapping to critical sub-networks moderating inflammation, immune function, and coagulation.

**Conclusions:**

We conclude that chronic regular smoking is associated with changes in peripheral mononuclear cell methylation signature which perturb inflammatory and immune function pathways and may contribute to increased vulnerability for complex illnesses with inflammatory components.

## Background

Smoking is the largest preventable cause of morbidity and mortality in the United States. It largely exerts these effects by increasing liability to complex disorders, such as cancer, chronic obstructive pulmonary disease (COPD), type 2 diabetes (T2DM) and obesity [1]. Smoking driven chronic diseases contribute to early death, disabilities, and strain the health care system [2]. Therefore, understanding the mechanism(s) through which smoking increases vulnerability to these disorders may establish new avenues for prevention or treatment of these complex disorders. Although some of the details remain unclear, one of the key mechanisms through which smoking may increase liability to these complex disorders is inflammation.

Although serological quantification of well characterized serum markers such as C-reactive protein (CRP) and interleukin 6 receptor (IL6R) may provide a partial understanding of inflammatory changes with respect to smoking, this approach provides limited comprehension of molecular perturbation at a genome-wide scale.

A surge in recent publications has suggested that smoking associated changes in DNA methylation may contribute to these perturbations. This surge began with sporadically published single gene studies that linked smoking to changes in *MAOA* promoter methylation as well as to an increased risk for coronary heart disease mediated through methylation changes at *F2RL3*[3,4]. But in the past two years, these more limited examinations have been joined by several genome-wide investigations that have identified a growing number of loci whose methylation status is associated to smoking. The initial attempt at a more systematic approach was reported by Breitling and colleagues who used the Illumina HumanMethylation 27K BeadChip to probe DNA from peripheral mononuclear cell pellets and identified several candidate loci including *F2RL3* (cg03636183), *GPR15* (cg19859270) and *ORAI2* (cg02564523) [5]. The first truly genome-wide results using the then newly introduced Illumina HumanMethylation 450K BeadChip were first reported by Monick and colleagues who studied methylation in lymphoblast and lung macrophage DNA and found a large number of loci with particular emphasis on differential methylation at the Aryl Hydrocarbon Receptor Repressor (*AHRR*) [6]. Six months later, using cord blood, Joubert and colleagues confirmed and extended these findings at *AHRR* and further nominated *GF1* and *CY1A1* as genes affected by smoking status [7]. Finally, in a study just published, Zeilinger and colleagues identified a larger set of findings that confirmed the prior loci noted above and extended the gene list to include loci such as *HIVEP3* (cg15542713), and *CACNA1D* (cg15417641 and cg21188533) [8].

These genome-wide finding present a potential portal for a better integrated understanding of pathways through which smoking potentially accelerates disease states. Previous studies have established several smoking associated disease pathways. One such is the cyclooxygenase-2 (COX-2) pathway where the expression of COX-2 induced by smoking leads to an increase in prostaglandin E2 (PGE2) that mediates tumor progression and an increase in thromboxane A2 (TxA2), contributing to tumor growth [9]. The COX-2 pathway that has been shown to be involved in numerous smoking related cancers is only one in many similar pathways that could conceivably hold the key to designing new therapeutic interventions and in some instances aid in improving current medical care.

A more recently developed method of identifying pathways perturbed by smoking is by coalescing information from network biology and epigenetics, specifically DNA methylation. DNA methylation is a critical mediator between the genome and the environment. Although genome-wide DNA methylation analyses allow the identification of differentially methylated CpG sites and genes, there is growing evidence that genes do not function independently, but rather in networks. Hence, it is crucial to not only delineate individual loci affected by long-term smoking, but, in addition, use network theory to translate that single locus methylation information into a more holistic understanding of the effects of smoking on the function of the proteome and the cell in general.

This global understanding is necessary to help understand the reasons why some individuals are affected more severely than others. Even though the rate of adults smoking is roughly equivalent in Whites and African Americans, African Americans disproportionately experience smoking associated medical comorbidities [10]. The reason for this disparity is not known but both genetic and sociological factors have been postulated. Epigenomic interrogation of this cohort would allow the disparity examination from a different perspective.

In prior studies, we have analyzed the relationship of smoking to peripheral mononuclear cell DNA methylation in young adult African American smokers [11]. To determine whether smoking and peripheral mononuclear cells may have a role in the systemic inflammation in long-term smokers, we conducted a study to examine the relationship of smoking to genome-wide DNA methylation in an independent and well characterized African American cohort who are older and have greater cumulative smoke exposure. In addition, we use a recently developed network based analysis technique to understand the effect of these smoking associated changes in DNA methylation on protein interaction networks. Our data suggest that regular chronic smoking is associated with highly significant differential methylation at a large number of loci including *AHRR* (cg05575921 and cg23576855) and *GPR15* (cg19859270). Furthermore, the bioinformatic analyses demonstrate enrichment of loci involved in regulation of inflammation, immune function and coagulation.

## Results

### Clinical characteristics of the cohort

The clinical characteristics of the 111 African American females from the FACHS project who participated in this study are given in Table 1. In brief, the subjects were middle-aged and tended to be severely obese. In this well characterized cohort, pack years was used as a quantitative measure of long-term cumulative smoking. Consistent with the high rate of medical comorbidities noted in this population, the average serum CRP levels (mg/l) were 5.0 in smokers (n = 50) and 2.9 in non-smokers (n = 61). The average serum IL6R levels (pg/ml) were 227 in smokers and 209 in non-smokers. The level of serum CRP (p < 0.05), but not the level of serum IL6R, was significantly higher in smokers.

**Table 1:** Clinical and demographic characteristics of female FACHS subjects participating in the genome-wide methylation studies

	**Smoker**	**Control**
Age	48.1 ± 7	48.7 ± 11
BMI	31.1 ± 8	35.3 ± 7
Smoking status	50	61
Current average cigarettes/day	5 ± 8	
^*^Smoker pack-years		
≥ ½ and < 10	17	
≥ 10 and < 20	7	
≥ 20	7	
CRP (mg/L)	5.02 ± 7.6	2.89 ± 3.2
IL6R (pg/mL)	227 ± 58	209 ± 56

*Total number of smoking individuals does not match the number of individuals accounted for under pack-years due to missing data points.

**Figure 1:**
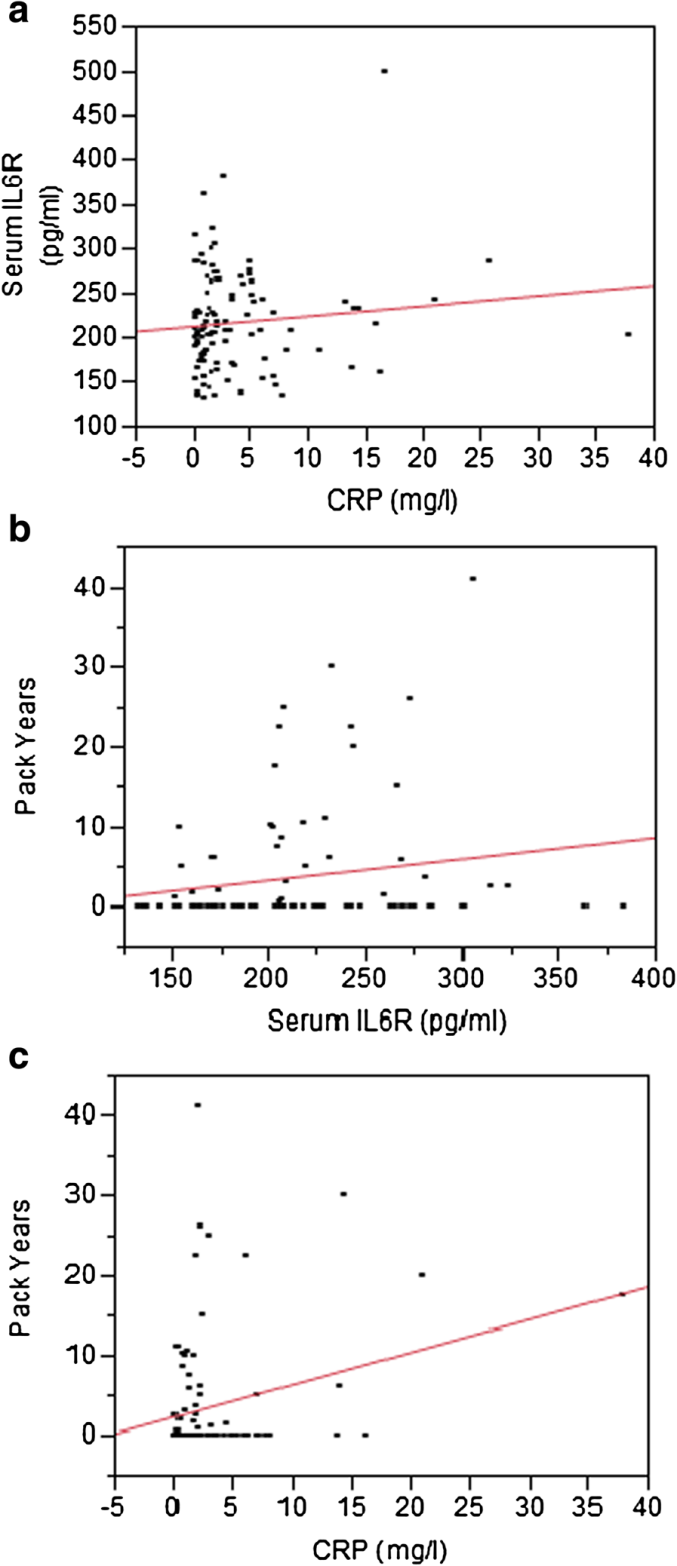
**Correlation between inflammatory cytokine levels and clinical characteristics of the FACHS female subjects.** Panel **a** illustrates the relationship between two serum inflammatory proteins, CRP and IL6R. Panels **b** and **c** represent the relationship between pack years and serum IL6R and CRP respectively.

As a first step of our analyses, we analyzed the relationship of the two serum inflammatory markers featured in this study, CRP and IL6R, to each other and to pack years. Figure 1 illustrates the results of those analyses. A positive correlation was present between CRP and IL6R. Significant positive association was only observed between pack years and CRP (p < 0.0092; Adj R^2^ = 0.064).

### Genome-wide DNA methylation analyses

We analyzed the genome-wide methylation data of peripheral mononuclear cell DNA using MethLAB with respect to smoking status, controlling for slide, plate and mixed cell population effects. This analysis was geared towards identifying differentially methylated CpG loci as a result of long-term smoking. Since inflammation is closely related to smoking, we speculated that the methylation of genes involved in inflammation would be perturbed. From the analysis, after genome-wide Benjamini-Hochberg correction, 910 probes remained significantly associated at the 0.05 level to smoking status. The 30 most significant CpG sites of this analysis are shown in Table 2 and all significant CpG sites are shown in Additional file 1. As hypothesized, the most significant gene noted was *GPR15* (cg19859270) which has been implicated in immune function as a gene that encodes for the G protein-coupled chemokine receptor for human immunodeficiency virus type 1 and 2.

**Table 2:** The thirty most significantly associated probes to smoking status after genome-wide correction

				**Average beta values**			
**Probe ID**	**Gene**	**Placement**	**Island status**	**Smokers**	**Non-smokers**	**T-test**	**Corrected p-value**
cg19859270	GPR15	1stExon		0.77	0.87	2.44E-25	1.19E-19
cg05575921	AHRR	Body	N_Shore	0.68	0.83	2.54E-24	6.17E-19
cg08672695			N_Shelf	0.65	0.48	4.58E-20	7.40E-15
cg23576855	AHRR	Body	N_Shore	0.52	0.71	2.78E-17	3.37E-12
cg02657160	CPOX	Body	N_Shore	0.77	0.83	4.09E-17	3.97E-12
cg21161138	AHRR	Body		0.62	0.70	5.74E-17	4.64E-12
cg18230367	RNASE4	TSS200	N_Shore	0.05	0.07	2.13E-16	1.48E-11
cg02319016	PAK2	5'UTR	S_Shelf	0.70	0.56	1.53E-15	9.27E-11
cg26607002	NOSTRIN	TSS200		0.68	0.63	4.31E-15	2.32E-10
cg04677326	C19orf28	TSS200	Island	0.17	0.15	4.98E-15	2.42E-10
cg01940273			Island	0.50	0.59	5.69E-15	2.51E-10
cg05457881	C6orf218	TSS1500		0.18	0.22	6.91E-15	2.79E-10
cg06126421				0.65	0.76	7.79E-15	2.91E-10
cg21566642			Island	0.38	0.48	8.62E-15	2.99E-10
cg13086586	PAICS	Body	S_Shore	0.17	0.23	3.26E-14	1.05E-09
cg15281724	TXLNB	Body		0.78	0.69	9.63E-14	2.92E-09
cg15645254	NAALAD2	Body		0.78	0.75	1.23E-13	3.51E-09
cg19111030	ANKRD53	TSS1500	N_Shore	0.17	0.20	1.96E-13	5.30E-09
cg09741592	HNRNPA1	Body	S_Shore	0.18	0.23	3.23E-13	8.25E-09
cg08528204	TMEM116	TSS1500	S_Shore	0.17	0.20	8.77E-13	2.07E-08
cg17391741			N_Shore	0.82	0.85	8.97E-13	2.07E-08
cg15614155			N_Shore	0.83	0.78	1.06E-12	2.34E-08
cg05916255	ABCC2	Body	N_Shore	0.85	0.82	1.17E-12	2.47E-08
cg25223391	UVRAG	Body		0.71	0.67	1.31E-12	2.52E-08
cg00736283	ASF1B	TSS200	Island	0.13	0.11	1.32E-12	2.52E-08
cg26703534	AHRR	Body	S_Shelf	0.62	0.68	1.35E-12	2.52E-08
cg16851858			N_Shelf	0.73	0.77	1.55E-12	2.80E-08
cg02521854			N_Shelf	0.13	0.11	1.69E-12	2.93E-08
cg15658543	CARD11	5'UTR		0.88	0.85	1.91E-12	3.20E-08
cg13789443	GALNT11	5'UTR	S_Shore	0.60	0.55	2.00E-12	3.24E-08

All average methylation values are non-log transformed beta-values. Island status refers to the position of the probe relative to the island. Classes include: 1) Island, 2) N (north) shore, 3) S (south) shore, 4) N (north) shelf, 5) S (south) shelf and 6) blank denoting that the probe does not map to an island.

Two other CpG probes, cg05575921 and cg23576855 (ranked 2^nd^ and 4^th^, respectively) were situated in the body of the aryl hydrocarbon receptor repressor (*AHRR*) gene. The protein encoded by this gene provides feedback inhibition of *AHR* activation of the xenobiotic pathway by several different mechanisms [12,13]. Our analyses indicate that on average, at these two loci, smokers tended to be hypomethylated. Moreover, our analyses are consistent with prior results of young adult African American smokers. In young adult African Americans, cg05575921 was also highly significantly affiliated to smoking and smokers tended to be hypomethylated. However, although hypomethylation was apparent at cg23576855 in both young and older smokers, this site was only significant with respect to long-term smoking and therefore could be a potential cumulative smoking biomarker.

### Validation of Illumina array findings

**Figure 2:**
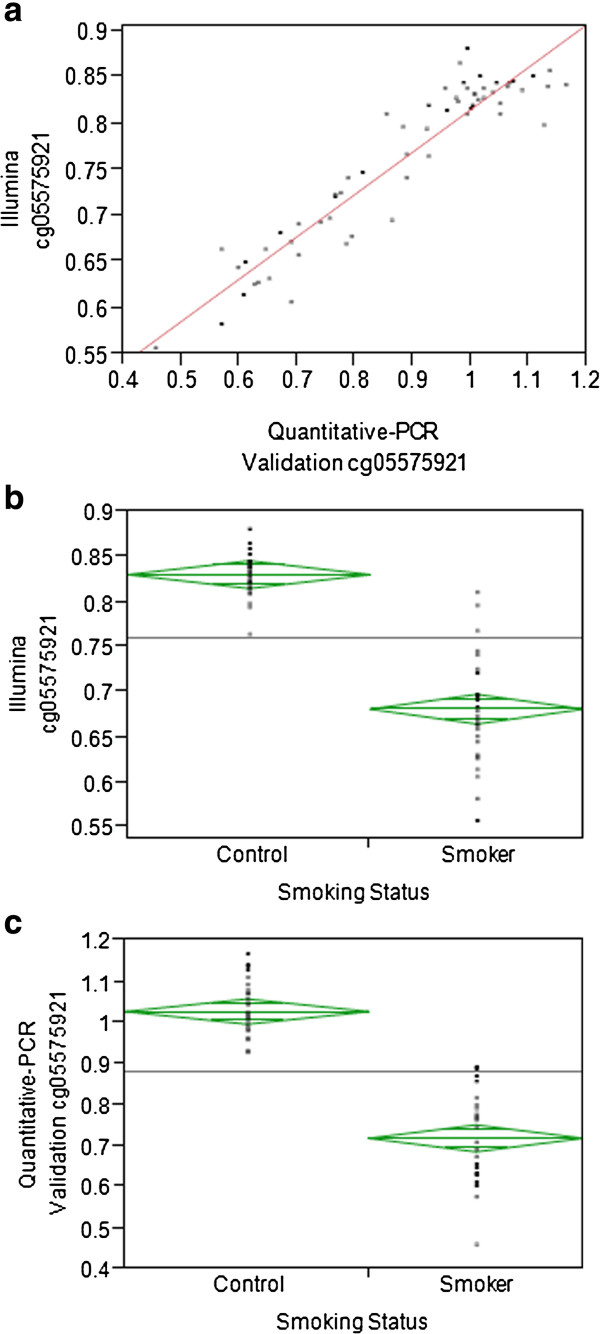
**Correlation between Illumina cg05575921 methylation and quantitative-PCR validation cg05575921 methylation and their respective association to smoking status.** Panel **a** illustrates the correlation between cg05575921 methylation from the Illumina platform and cg05575921 methylation from an independent quantitative-PCR validation (r = 0.94, p < 0.0001). Panels **b** and **c** depict the relationship between both independently obtained cg05575921 methylation level and smoking status.

Validation of genome-wide DNA methylation results is crucial, considering that the average beta values between smokers and non-smokers at numerous loci differed merely by about 0.1. Our previous study and this study indicate that cg05575921 as opposed to cg19859270 is not only sensitive to long-term smoking, but also to early smoking. Therefore, cg05575921 being the more consistently sensitive smoking biomarker was independently validated using quantitative-PCR on a subset of 62 African American females. The characteristics and methylation levels from both methods for cg05575921 for the individuals in this subset is given in Additional file 2. A strong correlation of 0.94 (p-value < 0.0001) between these two methylation detection methods was observed (Figure 2(a)). Furthermore, alongside the cg05575921 methylation level obtained via the Illumina platform, this independent approach also identified cg05575921 as a biomarker for smoking (ANOVA p-value < 0.0001 for both Illumina and quantitative-PCR as depicted in Figures 2(b) and 2(c), respectively).

### GoMiner analyses

We next analyzed the differential distribution of the methylation results using the traditional GoMiner algorithm which treats each probe as an independent assessment. These results are shown in Table 3. Surprisingly, after FDR correction, only 2 of the top 30 pathways were significant at the 0.05 level. Both of these pathways were associated with phospholipid transport.

**Table 3:** The top 30 differentially regulated gene ontology pathways from the GoMiner analysis

		**Genes**			
**GO category**	**Category name**	**Total**	**Changed**	**Log10 p-value**	**FDR**
GO:0004012	Phospholipid-translocating ATPase activity	15	6	-5.35	0.00
GO:0005548	Phospholipid transporter activity	29	7	-4.51	0.04
GO:0042954	Lipoprotein transporter activity	3	3		
GO:0015914	Phospholipid transport	33	7	-4.12	0.09
GO:0010876	Lipid localization	188	16	-3.36	0.37
GO:0016043	Cellular component organization	3231	136	-3.26	0.36
GO:0042623	ATPase activity coupled	276	20	-3.14	0.45
GO:0016887	ATPase activity	343	23	-3.06	0.44
GO:0003708	Retinoic acid receptor activity	7	3	-2.97	0.49
GO:0006869	Lipid transport	167	14	-2.95	0.45
GO:0045892	Negative regulation of transcription DNA-dependent	439	27	-2.92	0.42
GO:0005319	Lipid transporter activity	66	8	-2.89	0.39
GO:0051253	Negative regulation of RNA metabolic process	446	27	-2.82	0.42
GO:0071840	Cellular component organization or biogenesis	3341	137	-2.82	0.39
GO:0016817	Hydrolase activity acting on acid anhydrides	777	41	-2.80	0.38
GO:0042491	Auditory receptor cell differentiation	17	4	-2.73	0.42
GO:0042626	ATPase activity coupled to transmembrane movement of substances	103	10	-2.72	0.40
GO:0043492	ATPase activity coupled to movement of substances	104	10	-2.69	0.39
GO:0016820	Hydrolase activity acting on acid anhydrides catalyzing transmembrane movement of substances	105	10	-2.66	0.38
GO:0003727	Single-stranded RNA binding	29	5	-2.65	0.35
GO:0043954	Cellular component maintenance	29	5	-2.65	0.35
GO:0045668	Negative regulation of osteoblast differentiation	18	4	-2.63	0.35
GO:0016462	Pyrophosphatase activity	770	40	-2.62	0.34
GO:0005112	Notch binding	9	3	-2.61	0.35
GO:0016818	Hydrolase activity acting on acid anhydrides in phosphorus-containing anhydrides	773	40	-2.59	0.35
GO:0065007	Biological regulation	7226	267	-2.54	0.36
GO:0032504	Multicellular organism reproduction	514	29	-2.53	0.35
GO:0048609	Multicellular organismal reproductive process	514	29	-2.53	0.35
GO:0032502	Developmental process	3571	143	-2.53	0.34
GO:0004032	Aldehyde reductase activity	3	2		

### Protein-protein interactions: network analyses using the miPALM algorithm

Even though conventional genome-wide DNA methylation analyses yielded consistent identification of differentially methylated CpG loci at the *AHRR* gene, this analysis did not capture the connectivity of co-regulated genes as a result of long-term smoking. Hence, we employed an alternative approach for understanding the possible effects of smoking associated DNA methylation changes on cellular function by reducing the dimensionality of data and taking advantage of our understanding of the protein interactome. We used the miPALM algorithm which employs a greedy search strategy on weighted networks to re-analyze the genome-wide methylation data utilizing only the subset of DNA methylation data mapped to gene promoter regions.

**Figure 3:**
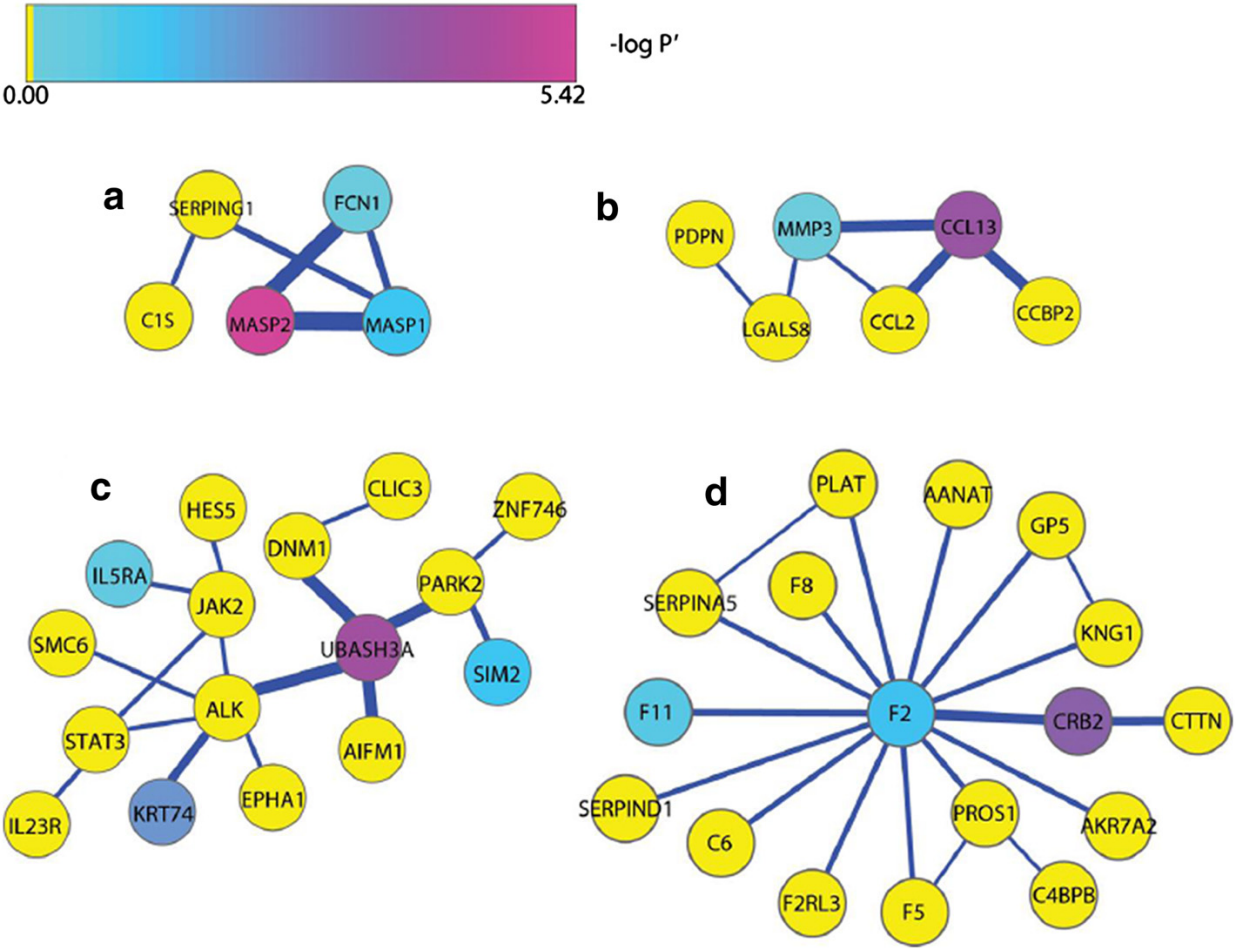
**Protein sub-networks identified by the miPALM algorithm and visualized using Cytoscape.** Strength of the interaction between two proteins is depicted by line width. The color of each node represents the negative log p-value from the t-test (see color bar insert) between smokers and non-smokers after correction for multiple comparisons (-log P’). Sub-network **(a)** and **(b)** consists of 5 proteins (p < 0.002) and 6 proteins (p < 0.008) respectively, enriched for immune function based on the BiNGO analysis. Sub-network **(c)** consists of 16 proteins (p < 0.05) enriched for the JAK-STAT signaling pathway based on the BiNGO analysis. Sub-network **(d)** consists of 17 proteins (p < 0.03) enriched for coagulation based on the BiNGO analysis.

Using this approach, smoking associated DNA methylation mapped to 10 significant protein sub-networks ranging from 5 (Figure 3(a)) to 24 (Additional file 3: Figure S1(b)) proteins with miPALM p-values ranging from p < 0.002 (Figure 3(a)) to p < 0.05 (Figure 3(c)). Figure 3 and Tables 4, 5, 6, 7 detail four sub-networks and the differential distribution of its constituent proteins with respect to Gene Ontology (GO) pathways. The first sub-network consists of 5 proteins, second consists of 6 proteins, third consists of 16 proteins and the fourth consists of 17 proteins. Consistent with our hypothesis, the BiNGO analysis demonstrated significant enrichment of proteins in the first and second sub-network to GO pathways involved in immune function (Tables 4 and 5), the third sub-network for the JAK-STAT signaling cascade (Table 6) and the fourth sub-network for coagulation (Table 7). BiNGO analysis of the other 6 sub-networks resulted in pathways being enriched for molecular transport (Additional file 4: Figure S2(a)), wounding (Additional file 4: Figures S2(b) and (c)), cell signaling (Additional file 3: Figure S1(a)), RNA processing (Additional file 3: Figure S1(b)), and central nervous system (Additional file 3: Figure S1(c)). Please see Additional file 3, Additional file 4, Additional file 5, Additional file 6, Additional file 7, Additional file 8, Additional file 9, Additional file 10 for complete details on these 6 sub-networks.

**Table 4:** **Top 10 pathways from BiNGO pathway analysis of protein sub-network depicted in Figure**3**(a)**

		**Genes**		
**GO category**	**Category name**	**Total**	**Changed**	**Corrected p-value**
GO:0002252	Immune effector process	129	5	6.23E-9
GO:0006956	Complement activation	40	4	1.09E-8
GO:0002541	Activation of plasma proteins in acute inflammatory response	41	4	1.09E-8
GO:0006959	Humoral immune response	77	4	1.07E-7
GO:0051605	Peptide maturation by peptide bond cleavage	81	4	1.07E-7
GO:0002526	Acute inflammatory response	89	4	1.31E-7
GO:0002253	Activation of immune response	96	4	1.53E-7
GO:0016485	Protein processing	105	4	1.92E-7
GO:0051604	Protein maturation	115	4	2.47E-7
GO:0050778	Positive regulation of immune response	146	4	5.84E-7

**Table 5:** **Top 10 pathways from BiNGO pathway analysis of protein sub-network depicted in Figure**3**(b)**

		**Genes**		
**GO category**	**Category name**	**Total**	**Changed**	**Corrected p-value**
GO:0042221	Response to chemical stimulus	1465	5	1.18E-3
GO:0042330	Taxis	169	3	1.18E-3
GO:0006935	Chemotaxis	169	3	1.18E-3
GO:0043200	Response to amino acid stimulus	20	2	1.18E-3
GO:0001101	Response to acid	27	2	1.74E-3
GO:0007626	Locomotory behavior	273	3	2.84E-3
GO:0014075	Response to amine stimulus	46	2	3.26E-3
GO:0006954	Inflammatory response	315	3	3.26E-3
GO:0040011	Locomotion	440	3	7.22E-3
GO:0010243	Response to organic nitrogen	77	2	7.22E-3

**Table 6:** **All pathways from BiNGO pathway analysis of protein sub-network depicted in Figure**3**(c)**

		**Genes**		
**GO category**	**Category name**	**Total**	**Changed**	**Corrected p-value**
GO:0060397	JAK-STAT cascade in growth hormone signaling pathway	4	2	3.49E-3
GO:0060396	Growth hormone receptor signaling pathway	8	2	5.41E-3
GO:0071378	Cellular response to growth hormone stimulus	8	2	5.41E-3
GO:0060416	Response to growth hormone stimulus	10	2	6.51E-3
GO:0007169	Transmembrane receptor protein tyrosine kinase signaling pathway	219	4	7.24E-3
GO:0007167	Enzyme linked receptor protein signaling pathway	346	4	3.51E-2
GO:0007259	JAK-STAT cascade	35	2	4.85E-2

**Table 7:** **Top 10 pathways from BiNGO pathway analysis of protein sub-network depicted in Figure**3**(d)**

		**Genes**		
**GO category**	**Category name**	**Total**	**Changed**	**Corrected p-value**
GO:0050878	Regulation of body fluid levels	146	11	4.69E-17
GO:0007596	Blood coagulation	103	10	9.69E-17
GO:0050817	Coagulation	103	10	9.69E-17
GO:0007599	Hemostasis	109	10	1.31E-16
GO:0042060	Wound healing	199	10	5.10E-14
GO:0009611	Response to wounding	541	12	2.17E-13
GO:0065008	Regulation of biological quality	1542	12	4.70E-8
GO:0006950	Response to stress	1773	12	2.10E-7
GO:0050896	Response to stimulus	3633	13	5.12E-5
GO:0002526	Acute inflammatory response	89	4	6.47E-5

## Discussion

In summary, we confirm prior observations demonstrating that on average, the level of CRP is higher in smokers than non-smokers, consistent with increased inflammation levels in smokers. We also extend findings by ourselves and others on the effect of smoking on peripheral mononuclear cell DNA methylation to show that continued smoking is associated with altered methylation signatures at *AHRR* and in pathways associated with immune, coagulation and CNS function.

The strength of the genome-wide findings with respect to the effects of smoking on DNA methylation indicates a need for a better longitudinal understanding of the response of the genome to tobacco smoke. In two consecutive prior studies of younger African American smokers with considerably less total cumulative tobacco smoke exposure, we demonstrated that the principal changes of the DNA methylation signature in response to smoking were in the xenobiotic response pathway regulated by the *AHRR* gene [11]. In contrast, in this group of African American female smokers, many of whom have been smoking for 25 or more years, the magnitude of the remodeling of the DNA methylation signature is significantly greater and extends to a number of other genes and pathways including those relevant to inflammation, immune, coagulation and CNS function. At the single gene level, the findings at *GFI1* (probe rank 24), *F2RL3* (probe rank 49) and *GPR15* (probe rank 1) serve to directly confirm prior findings by other investigators [5,7,14]. At a more integrated level, the implication of alterations of pathways involved in immune/inflammatory activities and coagulation (see Figure 3) is consistent with the known effects of smoking on risk for inflammatory related diseases and stroke [1,15].

Since there is a strong association between cigarette smoking and innate immunity, we hypothesized that networks enriching for immune system/inflammation would be generated. Two such networks are depicted in Figures 3(a) and (b) that highlight the potential role of peripheral mononuclear cells in moderating vulnerability to some but not all complex illnesses with inflammatory components. The BiNGO analysis of network 3(a) indicated that the proteins are involved in the response to acute inflammation and complement activation. Specifically, MASP2 with a differential methylation adjusted p-value of 3.78 × 10^-6^ is known to be a protease that activates the complement cascade, enabling the clearing of pathogens. It has been shown that MASP2 is also associated with the recurrence and survival rate of cancer (colorectal cancer in specific) [16]. Therefore, studying the effects of smoking in a weighted network approach allows us to speculate that patients with colorectal cancer may increase their survival chances and prevent the recurrence of cancer by avoiding smoking. In clinical settings, knowing smoking history could assist physicians in making better judgments of the efficacy of treatments.

Based on network 3(a), it can also be seen that MASP2 interacts strongly with MASP1 and FCN1. MASP1 is known to support MASP2 in the complement activation cascade [17] and this network validates that a strong interaction is present between these two serine proteases. MASP2, MASP1, and FCN1 play key roles in innate immunity. Aberrant methylation of these genes as a result of smoking could possibly impair an individual’s innate immunity, leading to the development, progression and recurrence of cancer. The network shown in Figure 3(b) is enriched for chemotaxis. Here, it can be noted that CCL13 with a differential methylation adjusted p-value of 0.00012 forms relatively strong interactions with MMP3, CCL2 and CCBP2. Again, similar to network 3(a), these interactions corroborate the role of smoking in the progression of inflammation.

Our work also builds on the prior work by Breitling and colleagues who showed the role of smoking associated differential methylation at coagulation factor II (thrombin) receptor like-3 (*F2RL3*) in moderating coronary artery disease, which also has a strong inflammatory component and whose presence is positively associated with CRP [3,18]. Furthermore, the network in Figure 3(d) that enriches for coagulation showed F2 as the central protein, forming interactions with other proteins including F11 and CRB2. The miPALM analyses which generated pathways with important implications demonstrate that the pathways involving IL6R and F2RL3 may not be the only important inflammatory genes/pathways operant in peripheral mononuclear cells and suggest the possibility that smoking associated DNA methylation may act through a variety of mechanisms to increase systemic inflammation and vulnerability to some illnesses with an inflammatory component.

The time frame in which these cells begin to experience smoking associated inflammatory changes in DNA methylation is not clear. In our prior examinations of young adult smokers, these changes were not evident on genome-wide basis. But these subjects were relatively young (19-22 years old) at the time of ascertainment and the cumulative smoking exposure of even the heaviest of the smokers was not as extensive as that the lightest of the current smokers [11]. Understanding when that shift to inflammatory processes occurs may be important to the allocation of smoking cessation resources. Although it is well established that in general smoking cessation decreases risk for adverse outcomes such as myocardial infarctions and cancer, this may not be true for all patients who quit smoking. According to the “pulmonary overflow” hypothesis, many of the inflammatory changes associated with smoking are not due to the direct effects of smoke but are rather secondary to the “spillover” of inflammatory cytokines from activated macrophages in the lung parenchyma. Because this inflammatory process tends to be self-perpetuating once it has been initiated, if “overflow” hypothesis is correct, stopping smoking in individuals with severe diseases may have little effect on subsequent inflammatory changes in the peripheral mononuclear cell compartment. If so, focusing smoking cessation resources on those smokers who do not have substantial diseases as evidenced by either peripheral or direct measures, may lead to more effective use of prevention dollars.

## Conclusions

In conclusion, we reiterate that smoking contributes to inflammation which is consistent with increase in the average serum inflammatory protein, CRP. We also demonstrate that methylation residues at the *AHRR* locus are sensitive to cumulative smoking and could be potential smoking biomarkers. However, the main finding in this well characterized cohort of long-term smokers is that differential methylation between smokers and non-smokers are key in delineating epigenetically contextual pathways and to identify highly connected networks that could be used for smoking related health diagnostics like cancer and coronary heart disease. We show the extensive effects of smoking on peripheral mononuclear cell DNA methylation on pathways involved in coagulation, CNS and immune function. Understanding aberrant methylation caused by smoking in a network setting provides an integrated stage for understanding complex diseases like cancer while designing potential therapies. Finally, smoking is an important confounder and should be included in future diagnostic models to accurately understand diseases.

## Methods

### Informed consent

The participants provided written consent to participate in this study. The written consent form, consent procedure, and protocols pertaining to this study were approved by the Institutional Review Board at the University of Iowa, the University of Georgia and Iowa State University.

### Human subjects

The subjects described in the current study are adult African American females from the states of Iowa and Georgia who participated in data and biomaterial collection during the most recent waves of the Family and Community Health Study (FACHS). The FACHS study is a longitudinal examination of factors affecting the health and health related outcomes of rural African American families [19]. These adults were identified because they had an 11 or 12-year-old child who resided in eligible recruitment area in either Iowa or Georgia. These geographical zones of eligibility varied with respect to socioeconomic status and were not selected for any other characteristic except relative enrichment in African American residents. Lists of these potential caretaker-offspring dyads living in these areas were then generated by community officials in Georgia and school officials in Iowa.

To recruit these individuals in the study, potential participant families were selected randomly from these lists and were then sent an introductory letter of invitation followed by a phone call. Families expressing interest in the study were then provided with a complete description of the study and if still willing, consented. The protocols and procedures pertaining to this study were approved by the Institutional Review Board at the University of Iowa, the University of Georgia and Iowa State University.

Each participant in the FACHS study is interviewed every two years in their homes with a battery of structured and semi-structured instruments that characterize the medical and psychological status of each subject and environment in which the subject is embedded. The data and biomaterials used in this study were collected during the most recent wave (Wave 10) of the project. As part of that examination, subjects were phlebotomized to provide biomaterials for the current study with a subset additionally being interviewed with the tobacco module from the Semi-Structured Assessment for the Genetics of Alcoholism-II (SSAGA-II) [20]. Individuals who were actively smoking were characterized as smokers and those denying any use of tobacco products were characterized as non-smokers.

### Genome-wide methylation profiling

For the current study, peripheral mononuclear cell DNA samples from FACHS subjects were assessed for genome-wide methylation status using our standard protocols [6,11]. In brief, DNA was prepared from ficoll purified peripheral mononuclear cell DNA pellets using a Qiagen (Valencia, CA) DNA Mini Kit. Measurement of genome-wide peripheral mononuclear cell DNA methylation status of FACHS participants was conducted using the Illumina (San Diego, CA) HumanMethylation450 Beadchip by the University of Minnesota Genome Center (Minneapolis, MN) using the protocol specified by the manufacturer [6]. This array contains 485,577 probes recognizing CpG positions of at least 20,216 known transcripts, potential transcripts or CpG islands. Subjects were randomly assigned to 12 sample “slides” with groups of 8 slides being bisulfite converted in a single batch. Four replicates of the same DNA were also included to monitor slide-to-slide and batch bisulfite conversion variability. The resulting data were inspected for complete bisulfite conversion and average beta values for each targeted CpG residue was determined using the Illumina Genome Studio Methylation Module, Version 3.2. The beta value at a CpG locus is the ratio between the intensity of the methylated probe to the sum of intensities of the methylated and unmethylated probes, also known as the total probe intensities. The resulting data was then processed using a custom PERL script to remove beta values with a detection p-value greater than 0.05.

### Accounting for mixed cell population

In order to account for mixed cell population in peripheral mononuclear cell mixture, a regression calibration approach similar to that developed by Houseman and colleagues was performed [21]. Since the method developed by Houseman and colleagues was based on the Illumina HumanMethylation 27K BeadChip, we instead utilized the Illumina HumanMethylation 450K BeadChip data of purified cells (CD4+ T cells, CD8+ T cells, CD14+ monocytes, CD19+ B cells and CD56+ Natural Killer cells) contributed by Reinius and colleagues (GEO database under accession number GSE35069) and performed a regression in MethLAB, Version 1.5 [22,23]. The Benjamini-Hochberg method at a 0.05 significance level was used for genome-wide correction. This resulted in 815 sites being significantly differentially methylated with respect to cell types. The top 100 sites were then imported into JMP Version 10 (SAS Institute, Cary, USA) and principal components analysis (PCA) was performed. Based on the Scree plot of this PCA, the first 5 factors were chosen to be included in downstream genome-wide analyses to control for signal as a result of mixed cell population.

### Analysis of genome-wide DNA methylation data

Genome-wide methylation data analyses were conducted using the R package, MethLAB, Version 1.5 as previously described [11]. Briefly, the genome-wide differential methylation between smokers and non-smokers were evaluated while controlling for slide, plate and mixed cell population effects. In MethLAB, the phenotype dependent differential methylation p-value at each CpG site is determined using a linear model [23]. Genome-wide false discovery rate was corrected using the method of Benjamini-Hochberg using the MethLAB default significance level of 0.05 [24].

### Validation of differential methylation at cg05575921

An independent validation of the differential methylation at cg05575921 was performed on a subset of 62 individuals using quantitative-PCR primer probe set obtained from Behavioral Diagnostics Inc (Iowa City, IA). Quantitative-PCR was performed with an ABI7900 (Life Technologies, New York) using provided reagents, internal controls and standard manufacturer protocols. Interpolation of internal control methylation was used to deduce percent methylation of each sample. Pearson correlation was performed to determine the correlation between the methylation level obtained via the Illumina platform and that obtained from the quantitative-PCR. Also, the relationship between cg05575921 methylation from the quantitative-PCR and smoking status was assessed using an analysis of variance (ANOVA) test.

### Protein network analyses

Protein sub-networks pertinent to smoking were identified using our previously described methods [25]. In brief, the genome-wide methylation data were first divided into bins based on the position of the CpG site with respect to the gene. We then focused the analyses on CpG probes that mapped to the promoter region of the gene. This is due to the known influence of promoter methylation on transcription [26]. This resulted in a total of 22,609 genes or potential gene transcripts with at least one CpG probe mapping to their putative promoter region. Using the data from all probes mapping to a given promoter region, two promoter methylation vectors were constructed for each gene; one for smokers and the other for non-smokers. Using the statistical programming language, R [27], for each gene, an unpaired t-test was performed between the two vectors resulting in p-values of differential methylation between smokers and non-smokers, that were adjusted for multiple comparisons using the Benjamini-Hochberg method [24].

Human protein-protein interaction data were downloaded from the iRefIndex database [28]. This database provides a comprehensive and non-redundant version of protein-protein interactions (PPI) available in several other primary interaction databases including BIND, BioGRID, CORUM, DIP, HPRD, IntAct, MINT, MPact, MPPI, and OPHID. The 22,609 genes were then compared to the non-redundant PPI that were extracted. There were 29,912 interactions that mapped to the list of genes. In order to construct an edge-weighted network, the subset of interactions alongside the adjusted p-values was used to calculate the PPI edge weights.

The following equation was used to calculate the edge weight between two genes:

$$w_{\mathrm{ij}}=\log\left(p{’}_{i}\times p{’}_{j}\right)/\log{\left(p{’}_{\min}\right)}^{2}$$

Where *w*_*ij*_ is the edge weight between genes i and j, *p’*_*i*_ is the adjusted p-value of the t-test between smokers and non-smokers for gene i, *p’*_*j*_ is the corresponding adjusted p-value for gene j, and *p’*_*min*_ is the minimum adjusted p-value among the 22,609 adjusted p-values calculated for the respective 22,609 genes.

Computed edge weights were assigned to their respective PPI and the resulting output was used to identify significant sub-networks using the miPALM algorithm implemented in JAVA. Briefly, this algorithm, which uses a greedy search method, identifies highly connected sub-networks using a novel graph theoretic measure, parametric local modularity [25,29].

Statistically significant sub-networks at the 0.05 significance level were imported into Cytoscape, a bioinformatics platform that allows visualization and analysis of biological networks [30]. The Cytoscape plugin BiNGO was used to determine Gene Ontology (GO) categories of statistically overrepresented genes [31]. In addition, the adjusted p-values of individual genes were imported as a node attribute and visualized as a color gradient. Edge weights were used as an edge attribute to represent the strength of interactions illustrated by the width of the lines connecting nodes.

### Enzyme linked immunosorbent assay (ELISA)

Sera were obtained using serum separator tubes. After centrifugation, sera were frozen at -80°C until use. ELISA assessments of CRP and IL6R serum levels were conducted using a DuoKit from R&D Systems (Minneapolis, USA) according to manufacturer’s recommendations.

### Regression analyses

The analyses of clinical and serological data were analyzed using the suite of general linear model algorithms (e.g. ANOVA, Bivariate, ordinal logistic regression) contained in JMP, Version 10 (SAS Institute, Cary, USA). Where indicated, the portion of the variance explained by the model tested is given by the Adjusted R square (Adj R^2^).

### Availability of supporting data

The Illumina Human Methylation 450K BeadChip data used in this study has been deposited to the Gene Expression Omnibus (GEO) database. The GEO accession number is GSE53045.

## Competing interests

The University of Iowa filed intellectual property right claims and has transferred some of those rights on some of the material related to this manuscript to Dr. Philibert. Specifically, the use of methylation at cg05575921 for the quantification of substance use is covered by U.S. Patent 8,637,652 while the use of other loci is patent pending. Dr. Philibert is also the Chief Scientific Officer for Behavioral Diagnostics, Inc. None of the other authors have any conflicts to disclose.

## Authors’ contributions

MVD, RAP and KT conceived and designed the study and experiments. MVD and RAP performed experiments and acquired data. All authors were involved in the analysis and interpretation of the data, drafting and revising the manuscript and read and approved the final version of the manuscript.

## Supplementary Material

Additional file 1 All 910 significant CpG sites with respect to smoking status after genome-wide correction. Click here for file

Additional file 2 Characteristics and average methylation levels at cg05575921 of a subset of 62 individuals used in an independent validation. Click here for file

Additional file 3: Figure S2 Protein sub-networks identified by the miPALM algorithm and visualized in Cytoscape. Click here for file

Additional file 4: Figure S1 Protein sub-networks identified by the miPALM algorithm and visualized in Cytoscape. Click here for file

Additional file 5 **Gene ontology pathways of Additional file**4**: Figure S1(a) identified by the Cytoscape plugin BiNGO.** Click here for file

Additional file 6 **Gene ontology pathways of Additional file**4**: Figure S1(b) identified by the Cytoscape plugin BiNGO.** Click here for file

Additional file 7 **Gene ontology pathways of Additional file**4**: Figure S1(c) identified by the Cytoscape plugin BiNGO.** Click here for file

Additional file 8 **Gene ontology pathways of Additional file**3**: Figure S2(a) identified by the Cytoscape plugin BiNGO.** Click here for file

Additional file 9 **Gene ontology pathways of Additional file**3**: Figure S2(b) identified by the Cytoscape plugin BiNGO.** Click here for file

Additional file 10 **Gene ontology pathways of Additional file**3**: Figure S2(c) identified by the Cytoscape plugin BiNGO.** Click here for file

## References

[B1] Centers for Disease Control Cigarette smoking among adults-United States 2006 Morbidity and Mortality Weekly Report 2007 56 1157 1161 17989644

[B2] Centers for Disease C, Prevention Smoking-attributable mortality, years of potential life lost, and productivity losses--United States, 2000-2004 MMWR Morb Mortal Wkly Rep 2008 57 1226 1228 19008791

[B3] Breitling LP Salzmann K Rothenbacher D Burwinkel B Brenner H Smoking, F2RL3 methylation, and prognosis in stable coronary heart disease Eur Heart J 2012 33 2841 2848 10.1093/eurheartj/ehs091 22511653

[B4] Philibert RA Beach SR Gunter TD Brody GH Madan A Gerrard M The effect of smoking on MAOA promoter methylation in DNA prepared from lymphoblasts and whole blood Am J Med Genet B Neuropsychiatr Genet 2010 153B 619 628 19777560 10.1002/ajmg.b.31031 PMC3694401

[B5] Breitling LP Yang R Korn B Burwinkel B Brenner H Tobacco-smoking-related differential DNA methylation: 27K discovery and replication American journal of human genetics 2011 88 450 457 10.1016/j.ajhg.2011.03.003 21457905 PMC3071918

[B6] Monick MM Beach SR Plume J Sears R Gerrard M Brody GH Philibert RA Coordinated changes in AHRR methylation in lymphoblasts and pulmonary macrophages from smokers Am J Med Genet B Neuropsychiatr Genet 2012 159B 141 151 10.1002/ajmg.b.32021 22232023 PMC3318996

[B7] Joubert BR Håberg SE Nilsen RM Wang X Vollset SE Murphy SK Huang Z Hoyo C Midttun O Cupul-Uicab LA 450K epigenome-wide scan identifies differential DNA methylation in newborns related to maternal smoking during pregnancy Environ Health Perspect 2012 120 1425 1431 10.1289/ehp.1205412 22851337 PMC3491949

[B8] Zeilinger S Kühnel B Klopp N Baurecht H Kleinschmidt A Gieger C Weidinger S Lattka E Adamski J Peters A Tobacco smoking leads to extensive genome-wide changes in DNA methylation PLoS One 2013 8 e63812 10.1371/journal.pone.0063812 23691101 PMC3656907

[B9] Huang RY Chen GG Cigarette smoking, cyclooxygenase-2 pathway and cancer Biochim Biophys Acta 2011 1815 158 169 21147199 10.1016/j.bbcan.2010.11.005

[B10] Max W Sung HY Tucker LY Stark B The disproportionate cost of smoking for African Americans in California Am J Public Health 2010 100 152 158 10.2105/AJPH.2008.149542 19965569 PMC2791258

[B11] Philibert RA Beach SR Brody GH Demethylation of the aryl hydrocarbon receptor repressor as a biomarker for nascent smokers Epigenetics 2012 7 1331 1338 10.4161/epi.22520 23070629 PMC3499333

[B12] Mimura J Ema M Sogawa K Fujii-Kuriyama Y Identification of a novel mechanism of regulation of Ah (dioxin) receptor function Genes Dev 1999 13 20 25 10.1101/gad.13.1.20 9887096 PMC316371

[B13] Oshima M Mimura J Yamamoto M Fujii-Kuriyama Y Molecular mechanism of transcriptional repression of AhR repressor involving ANKRA2, HDAC4, and HDAC5 Biochem Biophys Res Commun 2007 364 276 282 10.1016/j.bbrc.2007.09.131 17949687

[B14] Shenker NS Polidoro S van Veldhoven K Sacerdote C Ricceri F Birrell MA Belvisi MG Brown R Vineis P Flanagan JM Epigenome-wide association study in the European prospective investigation into cancer and nutrition (EPIC-Turin) identifies novel genetic loci associated with smoking Hum Mol Genet 2012 22 843 851 23175441 10.1093/hmg/dds488

[B15] Rose-John S Waetzig GH Scheller J Grötzinger J Seegert D The IL-6/sIL-6R complex as a novel target for therapeutic approaches Expert Opinion on Therapeutic Targets 2007 11 613 624 10.1517/14728222.11.5.613 17465721

[B16] Ytting H Christensen IJ Thiel S Jensenius JC Nielsen HJ Serum mannan-binding lectin-associated serine protease 2 levels in colorectal cancer: relation to recurrence and mortality Clin Cancer Res 2005 11 1441 1446 10.1158/1078-0432.CCR-04-1272 15746044

[B17] Dobó J Harmat V Beinrohr L Sebestyén E Závodszky P Gál P MASP-1, a promiscuous complement protease: structure of its catalytic region reveals the basis of its broad specificity J Immunol 2009 183 1207 1214 10.4049/jimmunol.0901141 19564340

[B18] Pearson TA Mensah GA Alexander RW Anderson JL Cannon RO Criqui M Fadl YY Fortmann SP Hong Y Myers GL Markers of inflammation and cardiovascular disease application to clinical and public health practice - a statement for healthcare professionals from the centers for disease control and prevention and the American Heart Association Circulation 2003 107 499 511 10.1161/01.CIR.0000052939.59093.45 12551878

[B19] Cutrona CE Russell DW Brown PA Clark LA Hessling RM Gardner KA Neighborhood context, personality, and stressful life events as predictors of depression among African American women Journal of Abnormal Psychology 2005 114 3 15 15709807 10.1037/0021-843X.114.1.3 PMC1913477

[B20] Bucholz KK Cadoret R Cloninger CR Dinwiddie SH Hesselbrock VM Nurnberger JI Jr Reich T Schmidt I Schuckit MA A new, semi-structured psychiatric interview for use in genetic linkage studies: a report on the reliability of the SSAGA J Stud Alcohol 1994 55 149 158 8189735 10.15288/jsa.1994.55.149

[B21] Houseman EA Accomando WP Koestler DC Christensen BC Marsit CJ Nelson HH Wiencke JK Kelsey KT DNA methylation arrays as surrogate measures of cell mixture distribution BMC Bioinformatics 2012 13 86 10.1186/1471-2105-13-86 22568884 PMC3532182

[B22] Reinius LE Acevedo N Joerink M Pershagen G Dahlén SE Greco D Söderhäll C Scheynius A Kere J Differential DNA methylation in purified human blood cells: implications for cell lineage and studies on disease susceptibility PLoS One 2012 7 e41361 10.1371/journal.pone.0041361 22848472 PMC3405143

[B23] Kilaru V Barfield RT Schroeder JW Smith AK Conneely KN MethLAB: a graphical user interface package for the analysis of array-based DNA methylation data Epigenetics 2012 7 225 229 10.4161/epi.7.3.19284 22430798 PMC3335946

[B24] Benjamini Y Hochberg Y Controlling the false discovery rate - a practical and powerful approach to multiple testing Journal of the Royal Statistical Society Series B-Methodological 1995 57 289 300

[B25] Kim J Gao L Tan K Multi-analyte network markers for tumor prognosis PLoS One 2012 7 e52973 10.1371/journal.pone.0052973 23300836 PMC3530467

[B26] Suzuki MM Bird A DNA methylation landscapes: provocative insights from epigenomics Nat Rev Genet 2008 9 465 476 10.1038/nrg2341 18463664

[B27] Team RC R: A language and environment for statistical computing 2012 Vienna, Austria: R Foundation for Statistical Computing

[B28] Razick S Magklaras G Donaldson IM iRefIndex: a consolidated protein interaction database with provenance Bmc Bioinformatics 2008 9 405 10.1186/1471-2105-9-405 18823568 PMC2573892

[B29] Kim J Tan K Discover protein complexes in protein-protein interaction networks using parametric local modularity BMC Bioinformatics 2010 11 521 10.1186/1471-2105-11-521 20958996 PMC2974752

[B30] Smoot ME Ono K Ruscheinski J Wang PL Ideker T Cytoscape 2.8: new features for data integration and network visualization Bioinformatics 2011 27 431 432 10.1093/bioinformatics/btq675 21149340 PMC3031041

[B31] Maere S Heymans K Kuiper M BiNGO: a cytoscape plugin to assess overrepresentation of gene ontology categories in biological networks Bioinformatics 2005 21 3448 3449 10.1093/bioinformatics/bti551 15972284

