# Supplementary material for: The effect of smoking on DNA methylation of peripheral blood mononuclear cells from African American women
Source: BMC Genomics. 2014 Feb 22;15:151. doi: 10.1186/1471-2164-15-151 (PMC3936875; doi:10.1186/1471-2164-15-151)
Supplement: Additional file 1 — All 910 significant CpG sites with respect to smoking status after genome-wide correction. [file 1471-2164-15-151-S1.docx]

## Additional File 1. Table S1. All 910 significantly associated probes to smoking status after genome-wide correction

|  |  |  |  | **Average beta values** | |  |  |
| --- | --- | --- | --- | --- | --- | --- | --- |
| **Probe ID** | **Gene** | **Placement** | **Island status** | **Smokers** | **Non-smokers** | **T-test** | **Corrected p-value** |
| cg19859270 | GPR15 | 1stExon |  | 0.77 | 0.87 | 2.44E-25 | 1.19E-19 |
| cg05575921 | AHRR | Body | N_Shore | 0.68 | 0.83 | 2.54E-24 | 6.17E-19 |
| cg08672695 | |  | N_Shelf | 0.65 | 0.48 | 4.58E-20 | 7.40E-15 |
| cg23576855 | AHRR | Body | N_Shore | 0.52 | 0.71 | 2.78E-17 | 3.37E-12 |
| cg02657160 | CPOX | Body | N_Shore | 0.77 | 0.83 | 4.09E-17 | 3.97E-12 |
| cg21161138 | AHRR | Body |  | 0.62 | 0.70 | 5.74E-17 | 4.64E-12 |
| cg18230367 | RNASE4 | TSS200 | N_Shore | 0.05 | 0.07 | 2.13E-16 | 1.48E-11 |
| cg02319016 | PAK2 | 5'UTR | S_Shelf | 0.70 | 0.56 | 1.53E-15 | 9.27E-11 |
| cg26607002 | NOSTRIN | TSS200 |  | 0.68 | 0.63 | 4.31E-15 | 2.32E-10 |
| cg04677326 | C19orf28 | TSS200 | Island | 0.17 | 0.15 | 4.98E-15 | 2.42E-10 |
| cg01940273 | |  | Island | 0.50 | 0.59 | 5.69E-15 | 2.51E-10 |
| cg05457881 | C6orf218 | TSS1500 |  | 0.18 | 0.22 | 6.91E-15 | 2.79E-10 |
| cg06126421 | |  |  | 0.65 | 0.76 | 7.79E-15 | 2.91E-10 |
| cg21566642 | |  | Island | 0.38 | 0.48 | 8.62E-15 | 2.99E-10 |
| cg13086586 | PAICS | Body | S_Shore | 0.17 | 0.23 | 3.26E-14 | 1.05E-09 |
| cg15281724 | TXLNB | Body |  | 0.78 | 0.69 | 9.63E-14 | 2.92E-09 |
| cg15645254 | NAALAD2 | Body |  | 0.78 | 0.75 | 1.23E-13 | 3.51E-09 |
| cg19111030 | ANKRD53 | TSS1500 | N_Shore | 0.17 | 0.20 | 1.96E-13 | 5.30E-09 |
| cg09741592 | HNRNPA1 | Body | S_Shore | 0.18 | 0.23 | 3.23E-13 | 8.25E-09 |
| cg08528204 | TMEM116 | TSS1500 | S_Shore | 0.17 | 0.20 | 8.77E-13 | 2.07E-08 |
| cg17391741 | |  | N_Shore | 0.82 | 0.85 | 8.97E-13 | 2.07E-08 |
| cg15614155 | |  | N_Shore | 0.83 | 0.78 | 1.06E-12 | 2.34E-08 |
| cg05916255 | ABCC2 | Body | N_Shore | 0.85 | 0.82 | 1.17E-12 | 2.47E-08 |
| cg25223391 | UVRAG | Body |  | 0.71 | 0.67 | 1.31E-12 | 2.52E-08 |
| cg00736283 | ASF1B | TSS200 | Island | 0.13 | 0.11 | 1.32E-12 | 2.52E-08 |
| cg26703534 | AHRR | Body | S_Shelf | 0.62 | 0.68 | 1.35E-12 | 2.52E-08 |
| cg16851858 | |  | N_Shelf | 0.73 | 0.77 | 1.55E-12 | 2.80E-08 |
| cg02521854 | |  | N_Shelf | 0.13 | 0.11 | 1.69E-12 | 2.93E-08 |
| cg15658543 | CARD11 | 5'UTR |  | 0.88 | 0.85 | 1.91E-12 | 3.20E-08 |
| cg13789443 | GALNT11 | 5'UTR | S_Shore | 0.60 | 0.55 | 2.00E-12 | 3.24E-08 |
| cg26381918 | C2orf64 | TSS1500 | S_Shore | 0.10 | 0.15 | 2.08E-12 | 3.26E-08 |
| cg09935388 | GFI1 | Body | Island | 0.70 | 0.82 | 2.18E-12 | 3.31E-08 |
| cg05951221 | |  | Island | 0.29 | 0.37 | 2.88E-12 | 4.23E-08 |
| cg25882591 | |  | N_Shore | 0.86 | 0.82 | 3.40E-12 | 4.86E-08 |
| cg18332146 | CTSC | TSS200 | Island | 0.24 | 0.22 | 4.84E-12 | 6.71E-08 |
| cg09174741 | THSD1 | TSS1500 | S_Shore | 0.83 | 0.79 | 6.34E-12 | 8.55E-08 |
| cg15105252 | |  |  | 0.38 | 0.42 | 7.72E-12 | 1.01E-07 |
| cg16105594 | MEF2C | 5'UTR | Island | 0.22 | 0.19 | 8.56E-12 | 1.07E-07 |
| cg18095109 | |  | Island | 0.14 | 0.12 | 8.61E-12 | 1.07E-07 |
| cg05944967 | NFATC1 | Body | Island | 0.93 | 0.91 | 9.07E-12 | 1.10E-07 |
| cg16830479 | ZC3H12B | TSS1500 |  | 0.64 | 0.55 | 1.06E-11 | 1.23E-07 |
| cg25677394 | HRH1 | TSS200 |  | 0.85 | 0.88 | 1.07E-11 | 1.23E-07 |
| cg18327772 | HMGB4 | 5'UTR |  | 0.76 | 0.80 | 1.14E-11 | 1.29E-07 |
| cg03333116 | RHBDF1 | Body | Island | 0.79 | 0.82 | 1.30E-11 | 1.43E-07 |
| cg10369313 | |  |  | 0.85 | 0.82 | 1.58E-11 | 1.71E-07 |
| cg26309498 | EDAR | TSS1500 |  | 0.69 | 0.57 | 1.69E-11 | 1.78E-07 |
| cg00389785 | |  | N_Shelf | 0.62 | 0.54 | 1.94E-11 | 2.00E-07 |
| cg09773647 | |  |  | 0.80 | 0.75 | 2.01E-11 | 2.03E-07 |
| cg05157912 | JDP2 | TSS200 | Island | 0.12 | 0.09 | 2.26E-11 | 2.24E-07 |
| cg11553667 | |  | S_Shelf | 0.66 | 0.55 | 2.32E-11 | 2.25E-07 |
| cg04120407 | KLHDC4 | Body |  | 0.97 | 0.96 | 2.44E-11 | 2.30E-07 |
| cg03636183 | F2RL3 | Body | N_Shore | 0.54 | 0.65 | 2.46E-11 | 2.30E-07 |
| cg22851561 | C14orf43 | 5'UTR |  | 0.37 | 0.44 | 2.62E-11 | 2.40E-07 |
| cg18450625 | EPB42 | TSS1500 |  | 0.70 | 0.78 | 2.71E-11 | 2.41E-07 |
| cg12636538 | SLC37A4 | 5'UTR | Island | 0.24 | 0.22 | 2.73E-11 | 2.41E-07 |
| cg24078767 | IFI35 | TSS1500 |  | 0.61 | 0.55 | 3.95E-11 | 3.39E-07 |
| cg14254999 | GPR55 | TSS1500 |  | 0.70 | 0.77 | 3.99E-11 | 3.39E-07 |
| cg19477192 | CCDC42B | Body | S_Shore | 0.89 | 0.85 | 4.22E-11 | 3.51E-07 |
| cg17417347 | GLYATL3 | 5'UTR |  | 0.83 | 0.89 | 4.27E-11 | 3.51E-07 |
| cg16906995 | |  |  | 0.72 | 0.67 | 4.60E-11 | 3.72E-07 |
| cg02995567 | TUBB2A | Body | Island | 0.10 | 0.12 | 4.86E-11 | 3.87E-07 |
| cg05062814 | C6orf64 | TSS200 | Island | 0.24 | 0.22 | 4.95E-11 | 3.87E-07 |
| cg24671951 | ATP8B2 | Body | S_Shore | 0.69 | 0.74 | 5.06E-11 | 3.90E-07 |
| cg04689145 | FRMD4B | Body |  | 0.82 | 0.78 | 5.36E-11 | 4.00E-07 |
| cg26687746 | |  |  | 0.55 | 0.50 | 5.43E-11 | 4.00E-07 |
| cg17414733 | ZNF429 | Body | Island | 0.37 | 0.45 | 5.43E-11 | 4.00E-07 |
| cg03991871 | AHRR | Body | N_Shore | 0.78 | 0.84 | 5.60E-11 | 4.01E-07 |
| cg08369295 | LRP5L | TSS1500 | S_Shelf | 0.97 | 0.96 | 5.62E-11 | 4.01E-07 |
| cg13397649 | AFG3L2 | 1stExon | Island | 0.18 | 0.16 | 6.12E-11 | 4.31E-07 |
| cg08026195 | C2orf27B | TSS1500 | Island | 0.17 | 0.22 | 7.10E-11 | 4.91E-07 |
| cg15514751 | CKS1B | Body | S_Shelf | 0.86 | 0.82 | 7.18E-11 | 4.91E-07 |
| cg16636692 | HIST1H2BK | TSS1500 | S_Shore | 0.10 | 0.13 | 7.84E-11 | 5.29E-07 |
| cg18731202 | TGFA | Body |  | 0.84 | 0.89 | 8.10E-11 | 5.39E-07 |
| cg27649653 | ZNF8 | TSS1500 | N_Shore | 0.23 | 0.28 | 8.55E-11 | 5.61E-07 |
| cg01344787 | |  |  | 0.70 | 0.65 | 8.88E-11 | 5.75E-07 |
| cg00746487 | |  |  | 0.88 | 0.85 | 9.11E-11 | 5.82E-07 |
| cg03811905 | |  | N_Shore | 0.74 | 0.77 | 1.00E-10 | 6.29E-07 |
| cg25808839 | LACTB | 1stExon | Island | 0.06 | 0.04 | 1.01E-10 | 6.29E-07 |
| cg21763952 | TCTN1 | TSS200 | Island | 0.17 | 0.15 | 1.02E-10 | 6.29E-07 |
| cg13440641 | DMTF1 | 5'UTR | S_Shore | 0.08 | 0.06 | 1.27E-10 | 7.64E-07 |
| cg22001073 | RICS | Body |  | 0.87 | 0.84 | 1.27E-10 | 7.64E-07 |
| cg10351795 | SLCO2A1 | Body | N_Shelf | 0.72 | 0.66 | 1.40E-10 | 8.29E-07 |
| cg00095276 | SLC12A7 | Body | N_Shore | 0.71 | 0.77 | 1.43E-10 | 8.38E-07 |
| cg06972908 | ITGAL | Body | S_Shelf | 0.63 | 0.70 | 1.45E-10 | 8.41E-07 |
| cg05874167 | |  |  | 0.63 | 0.59 | 1.50E-10 | 8.57E-07 |
| cg13668025 | SUCLG2 | TSS1500 | S_Shore | 0.27 | 0.31 | 1.52E-10 | 8.57E-07 |
| cg08621418 | HMGN3 | 5'UTR | Island | 0.09 | 0.13 | 1.71E-10 | 9.53E-07 |
| cg23267554 | |  |  | 0.84 | 0.78 | 1.79E-10 | 9.87E-07 |
| cg19550439 | ADAMTS6 | Body |  | 0.59 | 0.55 | 1.89E-10 | 1.01E-06 |
| cg22403154 | |  |  | 0.80 | 0.77 | 1.89E-10 | 1.01E-06 |
| cg03046325 | TNKS2 | Body | Island | 0.13 | 0.11 | 1.89E-10 | 1.01E-06 |
| cg14195606 | FAM190A | 5'UTR |  | 0.77 | 0.74 | 1.94E-10 | 1.02E-06 |
| cg01612443 | ATOH1 | TSS1500 | Island | 0.19 | 0.16 | 1.97E-10 | 1.03E-06 |
| cg14791530 | CTBS | TSS1500 | S_Shore | 0.73 | 0.79 | 2.04E-10 | 1.05E-06 |
| cg09552983 | |  |  | 0.80 | 0.76 | 2.12E-10 | 1.08E-06 |
| cg03482600 | |  |  | 0.79 | 0.83 | 2.13E-10 | 1.08E-06 |
| cg18968279 | TCF7L2 | Body |  | 0.84 | 0.87 | 2.22E-10 | 1.11E-06 |
| cg02641288 | IRX4 | TSS200 | Island | 0.36 | 0.34 | 2.28E-10 | 1.13E-06 |
| cg25354716 | CRTAP | Body | S_Shelf | 0.61 | 0.52 | 2.33E-10 | 1.14E-06 |
| cg01097768 | AHRR | Body |  | 0.49 | 0.57 | 2.44E-10 | 1.18E-06 |
| cg17632028 | PDE6B | TSS1500 | N_Shore | 0.83 | 0.85 | 2.49E-10 | 1.19E-06 |
| cg26546646 | ENSA | TSS200 | Island | 0.15 | 0.13 | 2.73E-10 | 1.30E-06 |
| cg18994438 | FMNL1 | Body | N_Shore | 0.80 | 0.84 | 2.90E-10 | 1.37E-06 |
| cg26674132 | ZNF559 | TSS1500 | N_Shore | 0.27 | 0.32 | 3.00E-10 | 1.40E-06 |
| cg23797200 | NKIRAS2 | Body | S_Shelf | 0.48 | 0.61 | 3.09E-10 | 1.43E-06 |
| cg01693063 | PRMT8 | TSS1500 | Island | 0.14 | 0.19 | 3.12E-10 | 1.43E-06 |
| cg11804350 | VWF | TSS1500 |  | 0.71 | 0.62 | 3.14E-10 | 1.43E-06 |
| cg16149164 | JAG2 | Body | Island | 0.57 | 0.63 | 3.26E-10 | 1.46E-06 |
| cg23003872 | |  |  | 0.72 | 0.69 | 3.32E-10 | 1.48E-06 |
| cg11580351 | SPATA9 | TSS1500 |  | 0.70 | 0.78 | 3.35E-10 | 1.48E-06 |
| cg22995176 | UPK3B | TSS1500 |  | 0.28 | 0.38 | 3.40E-10 | 1.49E-06 |
| cg18754985 | CLDND1 | Body | N_Shelf | 0.85 | 0.89 | 3.49E-10 | 1.51E-06 |
| cg05587870 | |  |  | 0.76 | 0.73 | 3.64E-10 | 1.56E-06 |
| cg04179819 | TAF3 | TSS200 | Island | 0.24 | 0.22 | 3.90E-10 | 1.64E-06 |
| cg19358594 | DFFA | 5'UTR | Island | 0.27 | 0.24 | 3.90E-10 | 1.64E-06 |
| cg17930737 | NOP58 | TSS1500 | N_Shore | 0.07 | 0.09 | 3.93E-10 | 1.64E-06 |
| cg10646962 | |  |  | 0.74 | 0.68 | 3.99E-10 | 1.64E-06 |
| cg24186711 | GUSBL1 | Body | N_Shore | 0.69 | 0.73 | 3.99E-10 | 1.64E-06 |
| cg12992443 | DLGAP4 | Body | S_Shelf | 0.72 | 0.76 | 4.13E-10 | 1.66E-06 |
| cg03384915 | SIN3B | Body | Island | 0.72 | 0.80 | 4.14E-10 | 1.66E-06 |
| cg27356115 | |  |  | 0.44 | 0.54 | 4.16E-10 | 1.66E-06 |
| cg26195710 | GNRH1 | TSS200 |  | 0.85 | 0.88 | 4.18E-10 | 1.66E-06 |
| cg10430189 | |  |  | 0.63 | 0.61 | 4.26E-10 | 1.67E-06 |
| cg04194664 | C17orf69 | Body |  | 0.77 | 0.84 | 4.26E-10 | 1.67E-06 |
| cg09606015 | ATP11B | Body | Island | 0.13 | 0.10 | 4.40E-10 | 1.71E-06 |
| cg04950839 | HAT1 | TSS200 | Island | 0.10 | 0.08 | 4.46E-10 | 1.72E-06 |
| cg22182287 | TTC15 | Body | Island | 0.89 | 0.91 | 4.60E-10 | 1.76E-06 |
| cg10094624 | ARL6IP1 | TSS1500 | Island | 0.13 | 0.11 | 4.69E-10 | 1.78E-06 |
| cg17267720 | PGBD4 | 1stExon | S_Shore | 0.60 | 0.53 | 4.80E-10 | 1.81E-06 |
| cg07404400 | TRIM41 | TSS1500 | Island | 0.17 | 0.15 | 4.98E-10 | 1.86E-06 |
| cg02578836 | |  |  | 0.70 | 0.67 | 5.48E-10 | 2.03E-06 |
| cg17971328 | SEMA4A | TSS1500 |  | 0.73 | 0.79 | 5.52E-10 | 2.03E-06 |
| cg01899089 | AHRR | Body | N_Shore | 0.46 | 0.52 | 5.63E-10 | 2.05E-06 |
| cg08828868 | DDX21 | TSS200 | N_Shore | 0.18 | 0.17 | 5.73E-10 | 2.08E-06 |
| cg10964388 | NTN4 | Body |  | 0.80 | 0.85 | 6.24E-10 | 2.23E-06 |
| cg11550862 | NCOR2 | Body | S_Shore | 0.97 | 0.96 | 6.27E-10 | 2.23E-06 |
| cg16709512 | |  |  | 0.88 | 0.86 | 6.30E-10 | 2.23E-06 |
| cg26057840 | |  | N_Shore | 0.73 | 0.79 | 6.57E-10 | 2.31E-06 |
| cg00155844 | HERC2 | Body |  | 0.76 | 0.74 | 6.91E-10 | 2.41E-06 |
| cg05920998 | HUS1B | 1stExon | Island | 0.95 | 0.94 | 7.52E-10 | 2.61E-06 |
| cg13675319 | |  | Island | 0.88 | 0.84 | 7.61E-10 | 2.62E-06 |
| cg06378498 | STAT3 | 5'UTR | Island | 0.19 | 0.16 | 7.97E-10 | 2.72E-06 |
| cg08360253 | PPP2CA | Body | Island | 0.10 | 0.08 | 8.21E-10 | 2.79E-06 |
| cg13324357 | |  |  | 0.92 | 0.90 | 8.32E-10 | 2.80E-06 |
| cg24793014 | SNORA59B | TSS1500 |  | 0.83 | 0.86 | 8.37E-10 | 2.80E-06 |
| cg21720999 | |  |  | 0.86 | 0.84 | 8.50E-10 | 2.83E-06 |
| cg06936779 | PIP5K1A | 5'UTR | Island | 0.30 | 0.39 | 8.80E-10 | 2.91E-06 |
| cg26270695 | CRABP1 | 3'UTR |  | 0.79 | 0.84 | 9.01E-10 | 2.95E-06 |
| cg23367119 | C14orf43 | 5'UTR | S_Shelf | 0.72 | 0.76 | 9.45E-10 | 3.08E-06 |
| cg23611710 | |  |  | 0.77 | 0.82 | 9.51E-10 | 3.08E-06 |
| cg19716125 | |  |  | 0.86 | 0.82 | 9.77E-10 | 3.13E-06 |
| cg13576178 | ZNF324B | TSS200 | Island | 0.17 | 0.15 | 9.79E-10 | 3.13E-06 |
| cg21385983 | PVALB | TSS200 | S_Shelf | 0.66 | 0.70 | 9.92E-10 | 3.14E-06 |
| cg10588834 | AUTS2 | Body |  | 0.79 | 0.83 | 9.97E-10 | 3.14E-06 |
| cg14941559 | NCK1 | 5'UTR |  | 0.81 | 0.78 | 1.02E-09 | 3.19E-06 |
| cg08866608 | |  | Island | 0.09 | 0.06 | 1.07E-09 | 3.32E-06 |
| cg15010854 | BAIAP2 | TSS1500 | N_Shore | 0.12 | 0.16 | 1.08E-09 | 3.34E-06 |
| cg25362525 | |  | Island | 0.55 | 0.54 | 1.09E-09 | 3.34E-06 |
| cg26456259 | |  |  | 0.69 | 0.75 | 1.10E-09 | 3.34E-06 |
| cg21898708 | C6orf48 | TSS1500 | N_Shore | 0.29 | 0.32 | 1.10E-09 | 3.34E-06 |
| cg19243391 | EIF4B | Body | Island | 0.13 | 0.10 | 1.12E-09 | 3.36E-06 |
| cg17512382 | |  |  | 0.89 | 0.87 | 1.12E-09 | 3.36E-06 |
| cg22094163 | KDM3A | Body |  | 0.64 | 0.71 | 1.13E-09 | 3.36E-06 |
| cg17731547 | COL23A1 | Body |  | 0.67 | 0.72 | 1.21E-09 | 3.57E-06 |
| cg27622633 | USP53 | TSS200 | Island | 0.13 | 0.11 | 1.22E-09 | 3.60E-06 |
| cg21290290 | C1orf93 | 5'UTR | Island | 0.29 | 0.38 | 1.24E-09 | 3.60E-06 |
| cg01837661 | GNB5 | Body |  | 0.36 | 0.32 | 1.24E-09 | 3.60E-06 |
| cg14651082 | |  | S_Shore | 0.71 | 0.75 | 1.25E-09 | 3.60E-06 |
| cg05573133 | FYCO1 | TSS200 | Island | 0.18 | 0.16 | 1.26E-09 | 3.60E-06 |
| cg18655915 | |  |  | 0.67 | 0.71 | 1.26E-09 | 3.60E-06 |
| cg03603505 | CLIP1 | Body |  | 0.94 | 0.93 | 1.27E-09 | 3.62E-06 |
| cg25648203 | AHRR | Body |  | 0.74 | 0.79 | 1.30E-09 | 3.66E-06 |
| cg17917920 | |  |  | 0.84 | 0.81 | 1.31E-09 | 3.66E-06 |
| cg00690392 | ENAH | Body |  | 0.82 | 0.84 | 1.31E-09 | 3.66E-06 |
| cg07065737 | |  | N_Shore | 0.78 | 0.81 | 1.32E-09 | 3.66E-06 |
| cg11168432 | MAEA | 3'UTR | Island | 0.98 | 0.97 | 1.33E-09 | 3.67E-06 |
| cg18474718 | ATM | Body |  | 0.76 | 0.72 | 1.37E-09 | 3.74E-06 |
| cg12880967 | MACROD1 | Body | Island | 0.15 | 0.13 | 1.37E-09 | 3.74E-06 |
| cg12950624 | RTN2 | 1stExon | Island | 0.07 | 0.05 | 1.38E-09 | 3.74E-06 |
| cg26182263 | SLC39A14 | TSS200 | Island | 0.22 | 0.19 | 1.39E-09 | 3.74E-06 |
| cg02247175 | |  | N_Shelf | 0.87 | 0.83 | 1.43E-09 | 3.83E-06 |
| cg17413252 | ENPP1 | Body | S_Shelf | 0.74 | 0.78 | 1.45E-09 | 3.88E-06 |
| cg01852611 | DSCR8 | TSS200 |  | 0.64 | 0.59 | 1.48E-09 | 3.92E-06 |
| cg12484845 | ZNF826 | 5'UTR | Island | 0.59 | 0.64 | 1.50E-09 | 3.96E-06 |
| cg02111705 | |  | N_Shore | 0.10 | 0.12 | 1.55E-09 | 4.06E-06 |
| cg11809157 | BBC3 | TSS1500 | Island | 0.06 | 0.05 | 1.56E-09 | 4.08E-06 |
| cg00833661 | KCNE1 | 5'UTR | N_Shore | 0.89 | 0.91 | 1.57E-09 | 4.08E-06 |
| cg22924269 | PHF11 | TSS1500 | N_Shore | 0.21 | 0.24 | 1.62E-09 | 4.16E-06 |
| cg08451992 | ARMC5 | TSS200 | Island | 0.07 | 0.06 | 1.62E-09 | 4.16E-06 |
| cg12317505 | CUBN | Body |  | 0.72 | 0.69 | 1.70E-09 | 4.33E-06 |
| cg04042800 | |  |  | 0.86 | 0.83 | 1.72E-09 | 4.38E-06 |
| cg11556164 | LRRN3 | 5'UTR |  | 0.71 | 0.76 | 1.75E-09 | 4.43E-06 |
| cg01638829 | |  |  | 0.93 | 0.91 | 1.79E-09 | 4.49E-06 |
| cg04031757 | |  |  | 0.21 | 0.19 | 1.80E-09 | 4.50E-06 |
| cg00642607 | |  |  | 0.83 | 0.86 | 1.81E-09 | 4.50E-06 |
| cg08283318 | ETFB | TSS1500 |  | 0.66 | 0.62 | 1.82E-09 | 4.50E-06 |
| cg14905634 | TRHDE | 3'UTR |  | 0.55 | 0.64 | 1.84E-09 | 4.50E-06 |
| cg21249729 | C9orf116 | TSS1500 | Island | 0.41 | 0.47 | 1.84E-09 | 4.50E-06 |
| cg02671915 | MAL2 | Body |  | 0.78 | 0.75 | 1.84E-09 | 4.50E-06 |
| cg09011231 | NCRNA00164 | TSS200 | Island | 0.63 | 0.68 | 1.90E-09 | 4.58E-06 |
| cg05620791 | |  |  | 0.87 | 0.84 | 1.91E-09 | 4.58E-06 |
| cg11140305 | SH3BP5L | Body | Island | 0.99 | 0.98 | 1.91E-09 | 4.58E-06 |
| cg26311995 | TRIM26 | Body |  | 0.69 | 0.74 | 1.92E-09 | 4.58E-06 |
| cg20891060 | |  | N_Shelf | 0.63 | 0.58 | 1.93E-09 | 4.58E-06 |
| cg21450008 | |  | N_Shore | 0.24 | 0.22 | 1.93E-09 | 4.58E-06 |
| cg07972458 | |  |  | 0.42 | 0.48 | 2.00E-09 | 4.72E-06 |
| cg02392575 | UHRF1BP1L | TSS200 | S_Shore | 0.21 | 0.19 | 2.02E-09 | 4.72E-06 |
| cg08531017 | DSCAML1 | Body | Island | 0.81 | 0.85 | 2.03E-09 | 4.72E-06 |
| cg02171500 | CHKA | Body | Island | 0.28 | 0.26 | 2.03E-09 | 4.72E-06 |
| cg21389924 | |  |  | 0.68 | 0.64 | 2.04E-09 | 4.72E-06 |
| cg11843502 | PER3 | TSS1500 | Island | 0.18 | 0.16 | 2.07E-09 | 4.77E-06 |
| cg07334509 | STAU2 | Body | Island | 0.94 | 0.92 | 2.15E-09 | 4.92E-06 |
| cg22225065 | C1orf57 | Body | S_Shore | 0.09 | 0.08 | 2.16E-09 | 4.92E-06 |
| cg02720697 | NFIC | TSS1500 | Island | 0.05 | 0.02 | 2.18E-09 | 4.93E-06 |
| cg27528104 | LOC100128023 | TSS1500 |  | 0.82 | 0.87 | 2.18E-09 | 4.93E-06 |
| cg22496377 | SHF | 5'UTR | S_Shore | 0.27 | 0.25 | 2.22E-09 | 4.99E-06 |
| cg11565042 | GPR45 | 1stExon | Island | 0.90 | 0.87 | 2.24E-09 | 5.00E-06 |
| cg24518943 | FAM135A | 5'UTR | Island | 0.12 | 0.16 | 2.25E-09 | 5.00E-06 |
| cg23916896 | AHRR | Body | N_Shore | 0.20 | 0.26 | 2.31E-09 | 5.11E-06 |
| cg18801567 | PDCD10 | 5'UTR | N_Shelf | 0.78 | 0.82 | 2.37E-09 | 5.23E-06 |
| cg05347948 | |  | S_Shelf | 0.79 | 0.74 | 2.38E-09 | 5.23E-06 |
| cg18016288 | ABCC4 | Body |  | 0.45 | 0.53 | 2.40E-09 | 5.24E-06 |
| cg16922869 | H1FOO | TSS1500 |  | 0.75 | 0.80 | 2.41E-09 | 5.24E-06 |
| cg11206312 | FGF8 | Body | N_Shore | 0.18 | 0.16 | 2.45E-09 | 5.31E-06 |
| cg19213194 | CUX1 | Body |  | 0.96 | 0.95 | 2.49E-09 | 5.38E-06 |
| cg04885881 | |  | S_Shelf | 0.35 | 0.43 | 2.54E-09 | 5.46E-06 |
| cg08668662 | PDRG1 | TSS200 | Island | 0.12 | 0.10 | 2.58E-09 | 5.52E-06 |
| cg08750493 | ZNF204P | Body | N_Shelf | 0.88 | 0.90 | 2.59E-09 | 5.52E-06 |
| cg04140754 | ATP6V0A2 | TSS1500 | N_Shore | 0.27 | 0.31 | 2.62E-09 | 5.56E-06 |
| cg14719752 | |  | Island | 0.97 | 0.96 | 2.68E-09 | 5.64E-06 |
| cg08488494 | ZNF365 | 5'UTR |  | 0.78 | 0.74 | 2.70E-09 | 5.64E-06 |
| cg16812288 | |  |  | 0.74 | 0.70 | 2.70E-09 | 5.64E-06 |
| cg24301350 | C2orf69 | 1stExon | Island | 0.24 | 0.20 | 2.71E-09 | 5.64E-06 |
| cg24311704 | MUC21 | 1stExon |  | 0.68 | 0.73 | 2.72E-09 | 5.64E-06 |
| cg23987549 | PMEPA1 | TSS200 | S_Shore | 0.14 | 0.10 | 2.74E-09 | 5.66E-06 |
| cg06438056 | AK2 | 1stExon | Island | 0.17 | 0.25 | 2.78E-09 | 5.70E-06 |
| cg21192376 | GPR137C | 1stExon | Island | 0.24 | 0.30 | 2.78E-09 | 5.70E-06 |
| cg14743683 | PTPRN2 | Body |  | 0.82 | 0.84 | 2.80E-09 | 5.71E-06 |
| cg19104471 | TOMM34 | TSS200 | Island | 0.05 | 0.04 | 2.84E-09 | 5.77E-06 |
| cg18864497 | SSR1 | 1stExon | Island | 0.12 | 0.10 | 2.89E-09 | 5.84E-06 |
| cg23674788 | KRT32 | 1stExon |  | 0.74 | 0.77 | 2.92E-09 | 5.87E-06 |
| cg09917026 | GNPTG | Body | Island | 0.97 | 0.96 | 2.93E-09 | 5.87E-06 |
| cg19110795 | UBXN7 | TSS1500 | Island | 0.11 | 0.09 | 3.04E-09 | 6.05E-06 |
| cg01938570 | ZBTB43 | 5'UTR | N_Shore | 0.85 | 0.87 | 3.04E-09 | 6.05E-06 |
| cg03467813 | FAM50B | 3'UTR | Island | 0.46 | 0.55 | 3.07E-09 | 6.09E-06 |
| cg18007641 | |  |  | 0.63 | 0.72 | 3.10E-09 | 6.09E-06 |
| cg13762887 | ZER1 | Body |  | 0.97 | 0.96 | 3.11E-09 | 6.09E-06 |
| cg04629194 | PDLIM1 | Body | Island | 0.10 | 0.09 | 3.11E-09 | 6.09E-06 |
| cg13566059 | TFDP3 | TSS200 |  | 0.74 | 0.66 | 3.13E-09 | 6.10E-06 |
| cg02569236 | ALDH1L1 | TSS1500 | S_Shore | 0.17 | 0.15 | 3.15E-09 | 6.12E-06 |
| cg00649216 | |  |  | 0.91 | 0.85 | 3.17E-09 | 6.13E-06 |
| cg06897921 | |  | Island | 0.08 | 0.06 | 3.29E-09 | 6.34E-06 |
| cg05079547 | NTM | TSS1500 |  | 0.62 | 0.69 | 3.37E-09 | 6.46E-06 |
| cg12416053 | ROBO2 | Body |  | 0.82 | 0.86 | 3.38E-09 | 6.46E-06 |
| cg22743003 | |  |  | 0.11 | 0.10 | 3.39E-09 | 6.46E-06 |
| cg05707844 | EIF2AK2 | 5'UTR | Island | 0.32 | 0.36 | 3.42E-09 | 6.49E-06 |
| cg07598331 | DMRTB1 | Body | Island | 0.79 | 0.82 | 3.45E-09 | 6.50E-06 |
| cg14628803 | |  |  | 0.78 | 0.74 | 3.47E-09 | 6.50E-06 |
| cg02292066 | C1orf93 | 5'UTR | Island | 0.16 | 0.20 | 3.47E-09 | 6.50E-06 |
| cg13461130 | PNPLA7 | Body | S_Shore | 0.97 | 0.96 | 3.50E-09 | 6.53E-06 |
| cg27097575 | ADARB2 | Body |  | 0.79 | 0.82 | 3.54E-09 | 6.56E-06 |
| cg24291974 | PLIN5 | Body | Island | 0.45 | 0.50 | 3.54E-09 | 6.56E-06 |
| cg21112148 | FBXL12 | Body | Island | 0.03 | 0.04 | 3.57E-09 | 6.59E-06 |
| cg02227015 | |  |  | 0.14 | 0.18 | 3.66E-09 | 6.72E-06 |
| cg18641329 | RCBTB2 | Body |  | 0.65 | 0.71 | 3.75E-09 | 6.87E-06 |
| cg08532673 | |  |  | 0.81 | 0.84 | 3.89E-09 | 7.10E-06 |
| cg27521571 | COMT | 5'UTR |  | 0.76 | 0.83 | 4.01E-09 | 7.27E-06 |
| cg19906093 | C2orf88 | 5'UTR | Island | 0.13 | 0.11 | 4.01E-09 | 7.27E-06 |
| cg10000843 | FANCI | TSS200 | Island | 0.09 | 0.08 | 4.05E-09 | 7.30E-06 |
| cg18055230 | FAM32A | Body | Island | 0.11 | 0.09 | 4.10E-09 | 7.38E-06 |
| cg07500957 | OTOR | 3'UTR |  | 0.78 | 0.82 | 4.17E-09 | 7.44E-06 |
| cg27573593 | PIK3CD | Body | S_Shelf | 0.96 | 0.94 | 4.17E-09 | 7.44E-06 |
| cg11213199 | MRPS24 | TSS200 | Island | 0.52 | 0.54 | 4.21E-09 | 7.49E-06 |
| cg14082938 | |  |  | 0.92 | 0.90 | 4.28E-09 | 7.57E-06 |
| cg22530977 | |  |  | 0.76 | 0.80 | 4.32E-09 | 7.62E-06 |
| cg24804436 | PPP2R5D | Body |  | 0.82 | 0.86 | 4.48E-09 | 7.88E-06 |
| cg21466736 | |  | S_Shore | 0.84 | 0.87 | 4.59E-09 | 8.02E-06 |
| cg21194937 | AP3D1 | TSS200 | Island | 0.08 | 0.06 | 4.60E-09 | 8.02E-06 |
| cg03662571 | SLC16A12 | 5'UTR |  | 0.82 | 0.85 | 4.65E-09 | 8.08E-06 |
| cg09999563 | AASDH | TSS200 | Island | 0.25 | 0.23 | 4.66E-09 | 8.08E-06 |
| cg16062877 | KSR1 | TSS1500 |  | 0.61 | 0.66 | 4.69E-09 | 8.10E-06 |
| cg09908764 | |  | N_Shelf | 0.36 | 0.42 | 4.75E-09 | 8.17E-06 |
| cg18874902 | CSNK1G3 | 1stExon | Island | 0.15 | 0.13 | 4.79E-09 | 8.22E-06 |
| cg12432846 | BCL10 | Body | Island | 0.12 | 0.18 | 4.85E-09 | 8.30E-06 |
| cg00727386 | |  |  | 0.72 | 0.68 | 4.89E-09 | 8.30E-06 |
| cg10216717 | TMEM132C | Body | N_Shore | 0.69 | 0.74 | 4.90E-09 | 8.30E-06 |
| cg11649016 | ITGA7 | Body |  | 0.85 | 0.87 | 4.91E-09 | 8.30E-06 |
| cg03168249 | KAZALD1 | Body | Island | 0.15 | 0.13 | 4.94E-09 | 8.32E-06 |
| cg03775901 | CNDP1 | 1stExon |  | 0.84 | 0.86 | 4.98E-09 | 8.36E-06 |
| cg21552822 | PDHX | 5'UTR | Island | 0.10 | 0.09 | 4.99E-09 | 8.36E-06 |
| cg18570553 | PRR15 | TSS200 | N_Shore | 0.12 | 0.10 | 5.11E-09 | 8.52E-06 |
| cg27607583 | TRAP1 | TSS200 | Island | 0.09 | 0.08 | 5.18E-09 | 8.62E-06 |
| cg10572794 | ABHD6 | 1stExon | Island | 0.09 | 0.08 | 5.22E-09 | 8.65E-06 |
| cg07120806 | WHSC1 | 5'UTR | Island | 0.14 | 0.19 | 5.31E-09 | 8.77E-06 |
| cg16063474 | STEAP3 | Body |  | 0.79 | 0.83 | 5.38E-09 | 8.85E-06 |
| cg26573518 | SLFN11 | TSS200 | Island | 0.12 | 0.10 | 5.42E-09 | 8.87E-06 |
| cg03862705 | NAT8B | TSS200 |  | 0.44 | 0.47 | 5.43E-09 | 8.87E-06 |
| cg18556005 | MGA | TSS200 | N_Shore | 0.06 | 0.08 | 5.45E-09 | 8.88E-06 |
| cg20755651 | SMOC2 | TSS1500 | Island | 0.12 | 0.08 | 5.47E-09 | 8.89E-06 |
| cg24533466 | SSBP3 | Body | Island | 0.90 | 0.92 | 5.65E-09 | 9.14E-06 |
| cg17054485 | |  | S_Shore | 0.93 | 0.91 | 5.67E-09 | 9.14E-06 |
| cg03761477 | |  | N_Shore | 0.28 | 0.25 | 5.73E-09 | 9.20E-06 |
| cg23098168 | CELSR2 | Body | Island | 0.98 | 0.97 | 5.82E-09 | 9.32E-06 |
| cg17456644 | KSR1 | 5'UTR |  | 0.76 | 0.79 | 5.84E-09 | 9.32E-06 |
| cg19017254 | TRPM4 | Body | S_Shore | 0.86 | 0.89 | 5.87E-09 | 9.35E-06 |
| cg06056514 | VARS | Body |  | 0.95 | 0.94 | 5.90E-09 | 9.36E-06 |
| cg18115235 | |  |  | 0.61 | 0.68 | 5.94E-09 | 9.39E-06 |
| cg16608652 | B3GALT2 | 5'UTR |  | 0.79 | 0.82 | 5.97E-09 | 9.41E-06 |
| cg22460590 | |  |  | 0.75 | 0.72 | 6.03E-09 | 9.47E-06 |
| cg05153748 | |  |  | 0.98 | 0.97 | 6.06E-09 | 9.49E-06 |
| cg26227186 | CNN3 | TSS1500 | S_Shore | 0.13 | 0.10 | 6.14E-09 | 9.59E-06 |
| cg24170784 | CABP7 | Body | S_Shelf | 0.75 | 0.78 | 6.27E-09 | 9.75E-06 |
| cg01442959 | HIST1H2AM | TSS1500 | Island | 0.11 | 0.14 | 6.30E-09 | 9.77E-06 |
| cg03508409 | PRR14 | TSS200 | Island | 0.10 | 0.13 | 6.38E-09 | 9.87E-06 |
| cg14515381 | VCP | TSS1500 | S_Shore | 0.77 | 0.82 | 6.56E-09 | 1.01E-05 |
| cg05377515 | |  |  | 0.54 | 0.51 | 6.58E-09 | 1.01E-05 |
| cg01746532 | HSPBP1 | TSS200 | Island | 0.08 | 0.11 | 6.64E-09 | 1.02E-05 |
| cg20353780 | TAOK3 | 3'UTR |  | 0.79 | 0.84 | 6.69E-09 | 1.02E-05 |
| cg08681117 | |  | Island | 0.76 | 0.79 | 6.72E-09 | 1.02E-05 |
| cg19572487 | RARA | 5'UTR | S_Shore | 0.37 | 0.44 | 6.73E-09 | 1.02E-05 |
| cg04995826 | API5 | TSS200 | Island | 0.21 | 0.19 | 6.84E-09 | 1.03E-05 |
| cg25538883 | MAPK4 | Body | S_Shore | 0.68 | 0.73 | 6.97E-09 | 1.05E-05 |
| cg16422343 | SFRS3 | Body | S_Shelf | 0.62 | 0.71 | 6.98E-09 | 1.05E-05 |
| cg26530713 | CRTC2 | Body | N_Shelf | 0.69 | 0.73 | 7.07E-09 | 1.06E-05 |
| cg07156182 | DIRC2 | TSS200 | Island | 0.10 | 0.13 | 7.53E-09 | 1.12E-05 |
| cg21364231 | LOC100128822 | TSS200 | Island | 0.14 | 0.16 | 7.56E-09 | 1.12E-05 |
| cg22459078 | |  |  | 0.84 | 0.81 | 7.60E-09 | 1.13E-05 |
| cg26947060 | ANKRD19 | TSS1500 | Island | 0.07 | 0.09 | 7.74E-09 | 1.14E-05 |
| cg23076299 | PCDH21 | TSS1500 | Island | 0.06 | 0.05 | 7.84E-09 | 1.15E-05 |
| cg16361253 | GPR111 | TSS200 |  | 0.69 | 0.72 | 7.84E-09 | 1.15E-05 |
| cg09387992 | |  |  | 0.63 | 0.61 | 7.88E-09 | 1.16E-05 |
| cg24859433 | |  |  | 0.77 | 0.81 | 7.95E-09 | 1.16E-05 |
| cg25155298 | TRERF1 | 5'UTR |  | 0.69 | 0.75 | 8.05E-09 | 1.17E-05 |
| cg01341751 | THRA | Body | Island | 0.97 | 0.95 | 8.05E-09 | 1.17E-05 |
| cg07991479 | KAT2A | Body | N_Shelf | 0.96 | 0.94 | 8.10E-09 | 1.17E-05 |
| cg21963656 | VARS | 1stExon | Island | 0.08 | 0.07 | 8.23E-09 | 1.19E-05 |
| cg24538512 | NFATC1 | Body | Island | 0.93 | 0.90 | 8.32E-09 | 1.20E-05 |
| cg08128444 | |  | S_Shelf | 0.47 | 0.42 | 8.42E-09 | 1.21E-05 |
| cg02693345 | |  |  | 0.88 | 0.86 | 8.43E-09 | 1.21E-05 |
| cg14170437 | CD300C | TSS1500 |  | 0.52 | 0.55 | 8.45E-09 | 1.21E-05 |
| cg06213807 | |  |  | 0.57 | 0.55 | 8.51E-09 | 1.21E-05 |
| cg11343534 | SETD1B | TSS200 | Island | 0.06 | 0.05 | 8.52E-09 | 1.21E-05 |
| cg03562528 | ASB2 | TSS200 |  | 0.76 | 0.80 | 8.58E-09 | 1.21E-05 |
| cg03244189 | TTTY14 | Body | Island | 0.22 | 0.15 | 8.60E-09 | 1.21E-05 |
| cg26698819 | |  |  | 0.72 | 0.77 | 8.60E-09 | 1.21E-05 |
| cg03038850 | |  |  | 0.82 | 0.85 | 8.65E-09 | 1.21E-05 |
| cg13958324 | ARID5B | Body |  | 0.80 | 0.84 | 8.69E-09 | 1.21E-05 |
| cg16650002 | C7orf57 | Body |  | 0.80 | 0.77 | 8.71E-09 | 1.22E-05 |
| cg20873046 | KDM4A | TSS200 | Island | 0.06 | 0.05 | 8.76E-09 | 1.22E-05 |
| cg01249134 | |  | N_Shelf | 0.41 | 0.36 | 8.82E-09 | 1.22E-05 |
| cg06431105 | ELL | Body | N_Shore | 0.94 | 0.92 | 8.90E-09 | 1.23E-05 |
| cg03525818 | |  | N_Shelf | 0.75 | 0.80 | 9.03E-09 | 1.24E-05 |
| cg21126306 | |  |  | 0.87 | 0.85 | 9.14E-09 | 1.26E-05 |
| cg00608779 | PRRX2 | Body | N_Shore | 0.71 | 0.76 | 9.19E-09 | 1.26E-05 |
| cg01418536 | USP32 | Body | N_Shelf | 0.81 | 0.78 | 9.27E-09 | 1.27E-05 |
| cg16727862 | ATP10A | Body |  | 0.16 | 0.14 | 9.30E-09 | 1.27E-05 |
| cg26061593 | |  | N_Shore | 0.70 | 0.74 | 9.47E-09 | 1.29E-05 |
| cg25411699 | ELMO1 | 5'UTR |  | 0.26 | 0.30 | 9.53E-09 | 1.29E-05 |
| cg18260823 | |  |  | 0.85 | 0.82 | 9.57E-09 | 1.29E-05 |
| cg18715665 | |  | Island | 0.93 | 0.91 | 9.63E-09 | 1.30E-05 |
| cg26319015 | |  | Island | 0.97 | 0.96 | 9.70E-09 | 1.30E-05 |
| cg11253957 | MYO18A | Body |  | 0.93 | 0.92 | 9.71E-09 | 1.30E-05 |
| cg18575346 | EP400 | Body |  | 0.97 | 0.96 | 9.81E-09 | 1.31E-05 |
| cg02264922 | B4GALNT1 | 5'UTR | Island | 0.09 | 0.08 | 9.82E-09 | 1.31E-05 |
| cg20778199 | |  |  | 0.58 | 0.66 | 9.87E-09 | 1.31E-05 |
| cg06557376 | MYH10 | Body |  | 0.94 | 0.92 | 9.92E-09 | 1.31E-05 |
| cg09043104 | |  |  | 0.75 | 0.79 | 9.92E-09 | 1.31E-05 |
| cg00486022 | KIFC3 | Body | S_Shore | 0.74 | 0.78 | 9.99E-09 | 1.32E-05 |
| cg15888699 | TIMM10 | TSS200 |  | 0.27 | 0.25 | 1.01E-08 | 1.32E-05 |
| cg02322048 | PIP5K1C | Body | N_Shore | 0.95 | 0.93 | 1.01E-08 | 1.32E-05 |
| cg16608731 | PIPOX | Body |  | 0.79 | 0.83 | 1.02E-08 | 1.33E-05 |
| cg01405684 | TBC1D10B | Body | S_Shelf | 0.92 | 0.90 | 1.03E-08 | 1.34E-05 |
| cg19655032 | |  |  | 0.75 | 0.78 | 1.04E-08 | 1.35E-05 |
| cg03651715 | AKAP12 | Body | N_Shore | 0.51 | 0.56 | 1.04E-08 | 1.35E-05 |
| cg18504937 | KLK9 | TSS1500 |  | 0.65 | 0.70 | 1.05E-08 | 1.35E-05 |
| cg10858195 | |  | N_Shelf | 0.64 | 0.61 | 1.08E-08 | 1.39E-05 |
| cg12090052 | TCP11 | TSS1500 | Island | 0.97 | 0.96 | 1.09E-08 | 1.40E-05 |
| cg23094080 | DGAT1 | 1stExon | Island | 0.18 | 0.16 | 1.11E-08 | 1.42E-05 |
| cg00712792 | SPIRE2 | Body | Island | 0.94 | 0.93 | 1.11E-08 | 1.43E-05 |
| cg14056470 | DYRK1B | Body | S_Shelf | 0.96 | 0.95 | 1.12E-08 | 1.43E-05 |
| cg04607032 | |  |  | 0.85 | 0.89 | 1.14E-08 | 1.45E-05 |
| cg06936402 | MMP20 | Body |  | 0.82 | 0.85 | 1.14E-08 | 1.45E-05 |
| cg14964274 | USH2A | 3'UTR |  | 0.78 | 0.82 | 1.15E-08 | 1.45E-05 |
| cg20668718 | JAKMIP2 | Body |  | 0.73 | 0.78 | 1.16E-08 | 1.46E-05 |
| cg24003539 | |  |  | 0.53 | 0.49 | 1.18E-08 | 1.49E-05 |
| cg22067527 | RFX2 | Body | Island | 0.89 | 0.91 | 1.18E-08 | 1.49E-05 |
| cg12145289 | PCNXL3 | Body | S_Shelf | 0.94 | 0.92 | 1.21E-08 | 1.52E-05 |
| cg25840536 | RBPMS2 | 3'UTR |  | 0.74 | 0.77 | 1.23E-08 | 1.54E-05 |
| cg05287481 | |  | Island | 0.67 | 0.71 | 1.24E-08 | 1.55E-05 |
| cg13412452 | DDX31 | Body | N_Shore | 0.28 | 0.33 | 1.25E-08 | 1.55E-05 |
| cg21078247 | CAMK2D | Body |  | 0.68 | 0.65 | 1.26E-08 | 1.56E-05 |
| cg26440142 | HLX | Body | Island | 0.85 | 0.87 | 1.26E-08 | 1.56E-05 |
| cg04715525 | ARGLU1 | TSS200 | Island | 0.07 | 0.08 | 1.26E-08 | 1.56E-05 |
| cg27401989 | SP1 | 5'UTR | Island | 0.11 | 0.14 | 1.27E-08 | 1.56E-05 |
| cg19925178 | |  |  | 0.72 | 0.69 | 1.30E-08 | 1.59E-05 |
| cg04167725 | GEMIN8 | Body |  | 0.84 | 0.80 | 1.30E-08 | 1.59E-05 |
| cg09580249 | RPH3AL | Body | N_Shelf | 0.79 | 0.82 | 1.31E-08 | 1.59E-05 |
| cg01111842 | PNPLA7 | 5'UTR | N_Shore | 0.10 | 0.12 | 1.31E-08 | 1.59E-05 |
| cg19614811 | GPR15 | TSS200 |  | 0.77 | 0.83 | 1.31E-08 | 1.59E-05 |
| cg09145256 | |  | Island | 0.24 | 0.22 | 1.31E-08 | 1.59E-05 |
| cg21571793 | FOXN3 | 5'UTR |  | 0.70 | 0.76 | 1.32E-08 | 1.59E-05 |
| cg09166536 | COL9A3 | Body | N_Shore | 0.72 | 0.78 | 1.32E-08 | 1.60E-05 |
| cg08676730 | PCTP | TSS200 | N_Shore | 0.08 | 0.12 | 1.33E-08 | 1.60E-05 |
| cg27345534 | PRB1 | TSS1500 |  | 0.73 | 0.78 | 1.36E-08 | 1.63E-05 |
| cg08826460 | LDLR | Body | Island | 0.15 | 0.13 | 1.36E-08 | 1.63E-05 |
| cg05274755 | NPAS3 | TSS200 |  | 0.18 | 0.15 | 1.37E-08 | 1.64E-05 |
| cg24315257 | |  |  | 0.73 | 0.69 | 1.38E-08 | 1.64E-05 |
| cg02917246 | GSC2 | TSS1500 | Island | 0.24 | 0.29 | 1.38E-08 | 1.64E-05 |
| cg03991106 | |  |  | 0.95 | 0.94 | 1.39E-08 | 1.64E-05 |
| cg04195702 | |  | N_Shore | 0.80 | 0.83 | 1.39E-08 | 1.64E-05 |
| cg14554244 | NOTCH1 | Body | N_Shore | 0.64 | 0.68 | 1.39E-08 | 1.64E-05 |
| cg06058681 | |  |  | 0.20 | 0.17 | 1.39E-08 | 1.64E-05 |
| cg14404146 | SLC24A2 | Body |  | 0.81 | 0.77 | 1.40E-08 | 1.64E-05 |
| cg27665449 | ANKRD53 | TSS1500 | N_Shore | 0.20 | 0.23 | 1.42E-08 | 1.66E-05 |
| cg27258878 | RBPMS | Body |  | 0.38 | 0.33 | 1.42E-08 | 1.66E-05 |
| cg08556938 | |  |  | 0.91 | 0.88 | 1.43E-08 | 1.67E-05 |
| cg19623519 | C1orf229 | TSS1500 | S_Shore | 0.78 | 0.80 | 1.44E-08 | 1.67E-05 |
| cg04729173 | |  |  | 0.73 | 0.77 | 1.44E-08 | 1.67E-05 |
| cg09583024 | RLTPR | Body | Island | 0.96 | 0.94 | 1.45E-08 | 1.68E-05 |
| cg20806296 | |  |  | 0.57 | 0.66 | 1.47E-08 | 1.70E-05 |
| cg03168749 | OR8B12 | TSS200 |  | 0.77 | 0.81 | 1.48E-08 | 1.71E-05 |
| cg26000722 | |  | S_Shelf | 0.08 | 0.11 | 1.49E-08 | 1.72E-05 |
| cg04294388 | |  | N_Shore | 0.71 | 0.75 | 1.50E-08 | 1.72E-05 |
| cg14923379 | FAM123B | 5'UTR | N_Shelf | 0.72 | 0.78 | 1.50E-08 | 1.72E-05 |
| cg19117365 | CLSTN2 | TSS200 | Island | 0.04 | 0.03 | 1.51E-08 | 1.73E-05 |
| cg05905475 | |  |  | 0.94 | 0.91 | 1.52E-08 | 1.73E-05 |
| cg04792227 | |  | Island | 0.25 | 0.17 | 1.52E-08 | 1.73E-05 |
| cg05832051 | MYADM | TSS200 | Island | 0.17 | 0.21 | 1.52E-08 | 1.73E-05 |
| cg15724941 | |  |  | 0.83 | 0.81 | 1.55E-08 | 1.75E-05 |
| cg09985739 | PCDH9 | Body |  | 0.74 | 0.82 | 1.56E-08 | 1.76E-05 |
| cg16582889 | GPT | TSS1500 | N_Shore | 0.75 | 0.78 | 1.56E-08 | 1.76E-05 |
| cg25051052 | SPIRE1 | 3'UTR |  | 0.81 | 0.85 | 1.57E-08 | 1.76E-05 |
| cg25575845 | |  |  | 0.79 | 0.83 | 1.57E-08 | 1.76E-05 |
| cg27248887 | EEF1B2 | Body | S_Shore | 0.36 | 0.41 | 1.58E-08 | 1.76E-05 |
| cg15894467 | C1orf190 | TSS1500 | N_Shore | 0.61 | 0.57 | 1.60E-08 | 1.78E-05 |
| cg09843155 | |  | N_Shelf | 0.52 | 0.44 | 1.60E-08 | 1.78E-05 |
| cg14191885 | |  |  | 0.69 | 0.74 | 1.61E-08 | 1.78E-05 |
| cg12584590 | TMEM233 | 3'UTR |  | 0.78 | 0.82 | 1.62E-08 | 1.80E-05 |
| cg18148726 | |  |  | 0.84 | 0.87 | 1.63E-08 | 1.80E-05 |
| cg19157327 | |  | N_Shelf | 0.76 | 0.79 | 1.64E-08 | 1.81E-05 |
| cg08914271 | |  | N_Shelf | 0.62 | 0.67 | 1.65E-08 | 1.82E-05 |
| cg14257543 | |  |  | 0.55 | 0.58 | 1.65E-08 | 1.82E-05 |
| cg01810593 | KIAA1967 | Body | S_Shore | 0.61 | 0.67 | 1.67E-08 | 1.83E-05 |
| cg22466850 | |  |  | 0.89 | 0.87 | 1.69E-08 | 1.85E-05 |
| cg10502231 | |  |  | 0.36 | 0.31 | 1.70E-08 | 1.85E-05 |
| cg12058781 | GBP4 | Body |  | 0.33 | 0.37 | 1.70E-08 | 1.85E-05 |
| cg26872588 | C1orf198 | Body |  | 0.82 | 0.84 | 1.71E-08 | 1.85E-05 |
| cg06968752 | |  | Island | 0.73 | 0.77 | 1.71E-08 | 1.85E-05 |
| cg00831247 | LOXL3 | 5'UTR | N_Shore | 0.75 | 0.78 | 1.71E-08 | 1.85E-05 |
| cg03881711 | |  |  | 0.89 | 0.84 | 1.72E-08 | 1.86E-05 |
| cg14595275 | C17orf72 | 1stExon |  | 0.09 | 0.08 | 1.78E-08 | 1.91E-05 |
| cg00124488 | BAT1 | 1stExon | Island | 0.30 | 0.28 | 1.79E-08 | 1.92E-05 |
| cg02724903 | TFDP1 | Body | Island | 0.86 | 0.84 | 1.80E-08 | 1.92E-05 |
| cg16226300 | RPS12 | Body | S_Shore | 0.14 | 0.19 | 1.80E-08 | 1.93E-05 |
| cg14242958 | KIAA1377 | TSS200 | Island | 0.11 | 0.09 | 1.81E-08 | 1.93E-05 |
| cg19189355 | DLL1 | Body | S_Shore | 0.77 | 0.79 | 1.82E-08 | 1.93E-05 |
| cg04573872 | CATSPER4 | TSS1500 |  | 0.76 | 0.80 | 1.82E-08 | 1.93E-05 |
| cg22092397 | |  | N_Shelf | 0.60 | 0.65 | 1.85E-08 | 1.96E-05 |
| cg22852353 | LMX1A | 3'UTR |  | 0.78 | 0.82 | 1.86E-08 | 1.96E-05 |
| cg02788938 | |  |  | 0.27 | 0.37 | 1.86E-08 | 1.96E-05 |
| cg14582009 | GLTSCR1 | Body | N_Shelf | 0.82 | 0.85 | 1.88E-08 | 1.97E-05 |
| cg12867237 | ACCS | TSS200 | Island | 0.17 | 0.15 | 1.88E-08 | 1.97E-05 |
| cg00421612 | |  | S_Shore | 0.63 | 0.69 | 1.89E-08 | 1.97E-05 |
| cg01810416 | |  | N_Shore | 0.89 | 0.92 | 1.89E-08 | 1.97E-05 |
| cg18123072 | CSNK1G3 | 5'UTR |  | 0.74 | 0.79 | 1.91E-08 | 2.00E-05 |
| cg01094121 | |  |  | 0.18 | 0.25 | 1.92E-08 | 2.00E-05 |
| cg05388492 | ESRRG | 5'UTR | Island | 0.14 | 0.10 | 1.93E-08 | 2.00E-05 |
| cg13750180 | ZNF815 | TSS1500 | N_Shore | 0.04 | 0.06 | 1.94E-08 | 2.01E-05 |
| cg00911962 | 6-Sep | Body |  | 0.73 | 0.80 | 1.96E-08 | 2.03E-05 |
| cg12340381 | |  |  | 0.59 | 0.56 | 1.98E-08 | 2.04E-05 |
| cg26487629 | |  | N_Shelf | 0.73 | 0.65 | 1.98E-08 | 2.04E-05 |
| cg00282216 | KIAA1688 | Body | Island | 0.61 | 0.59 | 1.98E-08 | 2.04E-05 |
| cg04717613 | |  | Island | 0.59 | 0.67 | 1.98E-08 | 2.04E-05 |
| cg08583001 | PVRL2 | TSS1500 | Island | 0.07 | 0.06 | 1.99E-08 | 2.04E-05 |
| cg18998938 | |  | S_Shelf | 0.32 | 0.39 | 2.00E-08 | 2.04E-05 |
| cg27060240 | |  |  | 0.86 | 0.88 | 2.00E-08 | 2.04E-05 |
| cg12920393 | HIST1H2BK | 3'UTR | Island | 0.12 | 0.15 | 2.01E-08 | 2.04E-05 |
| cg16475705 | UNC5C | Body | Island | 0.08 | 0.07 | 2.01E-08 | 2.04E-05 |
| cg03502236 | MUC2 | TSS200 |  | 0.71 | 0.74 | 2.06E-08 | 2.08E-05 |
| cg19069039 | |  | N_Shelf | 0.79 | 0.82 | 2.06E-08 | 2.08E-05 |
| cg19062112 | RCOR1 | TSS200 | Island | 0.10 | 0.09 | 2.07E-08 | 2.09E-05 |
| cg22397910 | CPSF3L | Body | Island | 0.05 | 0.04 | 2.07E-08 | 2.09E-05 |
| cg16602460 | PBX2 | Body |  | 0.72 | 0.76 | 2.09E-08 | 2.10E-05 |
| cg10888878 | |  | Island | 0.77 | 0.79 | 2.11E-08 | 2.11E-05 |
| cg21860285 | CPA6 | TSS1500 |  | 0.74 | 0.77 | 2.11E-08 | 2.11E-05 |
| cg07675184 | EMX2OS | Body | Island | 0.19 | 0.16 | 2.12E-08 | 2.12E-05 |
| cg10192196 | PSMB8 | 5'UTR | S_Shore | 0.30 | 0.28 | 2.13E-08 | 2.12E-05 |
| cg20805479 | CLK1 | 5'UTR | Island | 0.08 | 0.06 | 2.16E-08 | 2.15E-05 |
| cg05435065 | POR | TSS1500 | Island | 0.05 | 0.06 | 2.16E-08 | 2.15E-05 |
| cg16314263 | A2M | Body |  | 0.76 | 0.80 | 2.19E-08 | 2.17E-05 |
| cg21557724 | TSSC1 | Body | Island | 0.91 | 0.89 | 2.20E-08 | 2.17E-05 |
| cg06872036 | RASSF5 | Body | N_Shore | 0.73 | 0.77 | 2.21E-08 | 2.18E-05 |
| cg04636881 | |  |  | 0.92 | 0.89 | 2.22E-08 | 2.18E-05 |
| cg04880611 | MIR548H4 | Body |  | 0.81 | 0.83 | 2.22E-08 | 2.18E-05 |
| cg12989650 | ARHGEF15 | TSS1500 |  | 0.82 | 0.86 | 2.23E-08 | 2.19E-05 |
| cg06381350 | IRGC | TSS1500 | N_Shelf | 0.72 | 0.77 | 2.23E-08 | 2.19E-05 |
| cg11692477 | SLC40A1 | 5'UTR | Island | 0.07 | 0.06 | 2.25E-08 | 2.20E-05 |
| cg08076532 | |  |  | 0.70 | 0.67 | 2.26E-08 | 2.20E-05 |
| cg26433208 | CTPS | 5'UTR | Island | 0.12 | 0.11 | 2.26E-08 | 2.20E-05 |
| cg03792491 | GPR120 | TSS1500 | N_Shore | 0.83 | 0.86 | 2.26E-08 | 2.20E-05 |
| cg21694626 | |  |  | 0.78 | 0.75 | 2.27E-08 | 2.20E-05 |
| cg04433306 | UBE2H | 1stExon | Island | 0.12 | 0.15 | 2.28E-08 | 2.20E-05 |
| cg25842470 | |  | Island | 0.17 | 0.12 | 2.29E-08 | 2.21E-05 |
| cg07867325 | |  | Island | 0.51 | 0.46 | 2.31E-08 | 2.23E-05 |
| cg19212828 | |  |  | 0.57 | 0.52 | 2.32E-08 | 2.23E-05 |
| cg09333325 | KIF26A | Body |  | 0.82 | 0.84 | 2.34E-08 | 2.25E-05 |
| cg24162465 | |  |  | 0.88 | 0.86 | 2.35E-08 | 2.25E-05 |
| cg00828709 | C10orf71 | TSS200 |  | 0.80 | 0.85 | 2.36E-08 | 2.25E-05 |
| cg18865733 | DEFB125 | TSS1500 |  | 0.56 | 0.50 | 2.36E-08 | 2.25E-05 |
| cg21350392 | MTUS2 | 3'UTR | S_Shore | 0.98 | 0.97 | 2.37E-08 | 2.25E-05 |
| cg20529334 | SLCO1A2 | 5'UTR |  | 0.87 | 0.84 | 2.37E-08 | 2.25E-05 |
| cg00950473 | APCDD1L | Body | N_Shore | 0.66 | 0.72 | 2.38E-08 | 2.26E-05 |
| cg14313916 | |  |  | 0.74 | 0.70 | 2.39E-08 | 2.26E-05 |
| cg27589809 | CISH | TSS1500 | S_Shore | 0.61 | 0.67 | 2.39E-08 | 2.26E-05 |
| cg07078532 | SLC26A11 | 1stExon | Island | 0.09 | 0.08 | 2.41E-08 | 2.27E-05 |
| cg17114402 | ANKRD36 | TSS1500 | Island | 0.18 | 0.21 | 2.41E-08 | 2.27E-05 |
| cg08402107 | GSTO2 | 5'UTR | Island | 0.19 | 0.15 | 2.43E-08 | 2.27E-05 |
| cg03027241 | KCNG1 | 3'UTR | Island | 0.43 | 0.50 | 2.43E-08 | 2.27E-05 |
| cg20072001 | ASB7 | TSS1500 | Island | 0.25 | 0.23 | 2.43E-08 | 2.27E-05 |
| cg17063731 | |  |  | 0.47 | 0.45 | 2.43E-08 | 2.27E-05 |
| cg24651790 | |  |  | 0.78 | 0.74 | 2.43E-08 | 2.27E-05 |
| cg12617538 | |  | Island | 0.46 | 0.44 | 2.45E-08 | 2.27E-05 |
| cg06249109 | |  | N_Shore | 0.42 | 0.45 | 2.46E-08 | 2.28E-05 |
| cg07468782 | ZNF232 | 5'UTR | N_Shore | 0.66 | 0.71 | 2.47E-08 | 2.29E-05 |
| cg10703101 | AMAC1L3 | TSS200 | S_Shore | 0.77 | 0.80 | 2.48E-08 | 2.30E-05 |
| cg05586134 | PTCRA | Body | Island | 0.95 | 0.92 | 2.49E-08 | 2.30E-05 |
| cg17657594 | TRIM27 | TSS1500 | Island | 0.09 | 0.12 | 2.51E-08 | 2.31E-05 |
| cg05740739 | OR6B3 | TSS1500 |  | 0.75 | 0.81 | 2.51E-08 | 2.31E-05 |
| cg27379715 | |  |  | 0.57 | 0.66 | 2.55E-08 | 2.34E-05 |
| cg21599794 | XKR9 | Body |  | 0.80 | 0.77 | 2.58E-08 | 2.36E-05 |
| cg02882813 | CST5 | Body |  | 0.76 | 0.80 | 2.58E-08 | 2.36E-05 |
| cg04425458 | KCNQ3 | 1stExon | Island | 0.13 | 0.12 | 2.62E-08 | 2.39E-05 |
| cg25015290 | DCLRE1C | TSS200 | Island | 0.19 | 0.17 | 2.64E-08 | 2.40E-05 |
| cg26544188 | SNX29 | Body |  | 0.80 | 0.83 | 2.65E-08 | 2.41E-05 |
| cg02776750 | C12orf42 | TSS1500 | S_Shore | 0.84 | 0.86 | 2.66E-08 | 2.41E-05 |
| cg05938628 | MYO10 | TSS200 | N_Shore | 0.25 | 0.31 | 2.68E-08 | 2.43E-05 |
| cg20992503 | NACA2 | TSS200 |  | 0.86 | 0.83 | 2.69E-08 | 2.43E-05 |
| cg16775460 | TBC1D16 | Body | Island | 0.84 | 0.86 | 2.71E-08 | 2.44E-05 |
| cg16168311 | APOA1BP | Body | S_Shore | 0.18 | 0.22 | 2.71E-08 | 2.44E-05 |
| cg25910466 | XRN2 | 5'UTR | Island | 0.15 | 0.14 | 2.73E-08 | 2.45E-05 |
| cg18233942 | C1orf53 | Body | Island | 0.04 | 0.03 | 2.74E-08 | 2.46E-05 |
| cg21646084 | FAM49A | 5'UTR |  | 0.76 | 0.79 | 2.74E-08 | 2.46E-05 |
| cg12075928 | PTK2 | Body |  | 0.36 | 0.43 | 2.75E-08 | 2.46E-05 |
| cg23629792 | PRKCZ | Body | N_Shelf | 0.83 | 0.87 | 2.76E-08 | 2.46E-05 |
| cg15756928 | GFRAL | 3'UTR |  | 0.79 | 0.82 | 2.76E-08 | 2.46E-05 |
| cg18396789 | |  |  | 0.76 | 0.80 | 2.78E-08 | 2.46E-05 |
| cg22091132 | SEC11C | 1stExon | Island | 0.06 | 0.05 | 2.78E-08 | 2.46E-05 |
| cg01323766 | |  |  | 0.91 | 0.88 | 2.78E-08 | 2.46E-05 |
| cg09249637 | PQLC3 | Body |  | 0.65 | 0.67 | 2.78E-08 | 2.46E-05 |
| cg25867545 | LAMP3 | TSS200 | S_Shore | 0.16 | 0.18 | 2.79E-08 | 2.46E-05 |
| cg06897120 | LOC342346 | Body |  | 0.88 | 0.80 | 2.80E-08 | 2.46E-05 |
| cg08708209 | DUSP10 | TSS200 | Island | 0.13 | 0.11 | 2.81E-08 | 2.47E-05 |
| cg15267250 | |  | S_Shore | 0.73 | 0.76 | 2.82E-08 | 2.47E-05 |
| cg27167195 | PKP4 | 5'UTR |  | 0.81 | 0.77 | 2.82E-08 | 2.47E-05 |
| cg14768782 | KIAA1257 | TSS200 | Island | 0.18 | 0.16 | 2.83E-08 | 2.47E-05 |
| cg17451760 | LTBP3 | TSS1500 | S_Shore | 0.62 | 0.66 | 2.84E-08 | 2.48E-05 |
| cg18985133 | IDO2 | Body |  | 0.79 | 0.82 | 2.90E-08 | 2.52E-05 |
| cg09916030 | CLDN14 | TSS1500 |  | 0.79 | 0.82 | 2.91E-08 | 2.52E-05 |
| cg16098304 | GAL3ST2 | Body |  | 0.77 | 0.81 | 2.91E-08 | 2.52E-05 |
| cg15959715 | GAD2 | Body | Island | 0.13 | 0.11 | 2.92E-08 | 2.52E-05 |
| cg14840863 | COLEC11 | Body |  | 0.66 | 0.70 | 2.92E-08 | 2.52E-05 |
| cg11779204 | CTNND2 | Body |  | 0.74 | 0.78 | 2.93E-08 | 2.53E-05 |
| cg01720945 | PLEKHA6 | Body | Island | 0.98 | 0.97 | 2.96E-08 | 2.55E-05 |
| cg24070837 | ZNF382 | Body |  | 0.85 | 0.87 | 2.99E-08 | 2.57E-05 |
| cg09542690 | ZNF445 | TSS200 | Island | 0.21 | 0.19 | 3.02E-08 | 2.59E-05 |
| cg18334819 | |  |  | 0.77 | 0.81 | 3.03E-08 | 2.59E-05 |
| cg04758185 | PLK1 | Body |  | 0.96 | 0.95 | 3.03E-08 | 2.59E-05 |
| cg25457109 | |  |  | 0.83 | 0.85 | 3.04E-08 | 2.60E-05 |
| cg22010909 | ATP11A | Body | S_Shore | 0.96 | 0.95 | 3.05E-08 | 2.60E-05 |
| cg19023320 | IQSEC1 | Body | N_Shore | 0.76 | 0.79 | 3.06E-08 | 2.61E-05 |
| cg09106556 | ZNF302 | TSS1500 | Island | 0.08 | 0.10 | 3.07E-08 | 2.61E-05 |
| cg27110491 | UPP1 | 5'UTR | S_Shelf | 0.81 | 0.83 | 3.08E-08 | 2.61E-05 |
| cg02406531 | |  |  | 0.64 | 0.52 | 3.08E-08 | 2.61E-05 |
| cg07842327 | SKI | Body |  | 0.95 | 0.93 | 3.11E-08 | 2.62E-05 |
| cg19515468 | |  |  | 0.85 | 0.82 | 3.11E-08 | 2.62E-05 |
| cg02351018 | |  |  | 0.80 | 0.77 | 3.11E-08 | 2.62E-05 |
| cg05112617 | ARL13B | TSS200 | Island | 0.23 | 0.21 | 3.12E-08 | 2.63E-05 |
| cg10583473 | MICALL2 | Body | Island | 0.79 | 0.82 | 3.13E-08 | 2.63E-05 |
| cg21217129 | ADARB1 | 5'UTR | Island | 0.79 | 0.82 | 3.13E-08 | 2.63E-05 |
| cg24377560 | AFAP1 | Body |  | 0.46 | 0.41 | 3.15E-08 | 2.63E-05 |
| cg19910568 | DNAJC1 | Body |  | 0.97 | 0.95 | 3.15E-08 | 2.63E-05 |
| cg15777910 | TRPV1 | Body |  | 0.96 | 0.95 | 3.16E-08 | 2.63E-05 |
| cg06648782 | PACRG | Body |  | 0.83 | 0.86 | 3.17E-08 | 2.64E-05 |
| cg08257600 | ADCK4 | Body | S_Shelf | 0.66 | 0.73 | 3.17E-08 | 2.64E-05 |
| cg01731783 | C14orf43 | 5'UTR |  | 0.51 | 0.56 | 3.20E-08 | 2.65E-05 |
| cg20407796 | B3GNTL1 | Body | N_Shore | 0.78 | 0.82 | 3.20E-08 | 2.65E-05 |
| cg07387813 | RPL14 | Body | S_Shore | 0.13 | 0.17 | 3.24E-08 | 2.68E-05 |
| cg03421069 | PAN3 | Body |  | 0.47 | 0.43 | 3.25E-08 | 2.68E-05 |
| cg03065888 | CYC1 | TSS1500 | N_Shore | 0.72 | 0.64 | 3.26E-08 | 2.68E-05 |
| cg18040892 | SMARCA4 | Body | Island | 0.93 | 0.91 | 3.33E-08 | 2.73E-05 |
| cg24807761 | CALD1 | Body |  | 0.96 | 0.95 | 3.33E-08 | 2.73E-05 |
| cg24263998 | |  |  | 0.84 | 0.86 | 3.33E-08 | 2.73E-05 |
| cg08888410 | ZSCAN10 | Body | Island | 0.98 | 0.97 | 3.34E-08 | 2.73E-05 |
| cg12742432 | SYTL1 | Body | Island | 0.88 | 0.85 | 3.37E-08 | 2.75E-05 |
| cg01604210 | HMGCL | Body | S_Shore | 0.83 | 0.85 | 3.37E-08 | 2.75E-05 |
| cg09180820 | PRKCZ | Body | N_Shelf | 0.73 | 0.76 | 3.39E-08 | 2.76E-05 |
| cg10502232 | ATP6V0C | 5'UTR | Island | 0.03 | 0.01 | 3.40E-08 | 2.76E-05 |
| cg00498419 | MKI67 | TSS1500 | S_Shore | 0.12 | 0.15 | 3.40E-08 | 2.76E-05 |
| cg11870561 | |  |  | 0.76 | 0.79 | 3.41E-08 | 2.76E-05 |
| cg07437919 | SLC45A4 | Body | N_Shore | 0.36 | 0.45 | 3.45E-08 | 2.78E-05 |
| cg14303616 | RTP1 | Body | Island | 0.73 | 0.76 | 3.45E-08 | 2.78E-05 |
| cg19615731 | DIDO1 | 3'UTR |  | 0.80 | 0.83 | 3.45E-08 | 2.78E-05 |
| cg14065224 | VWA3B | Body |  | 0.88 | 0.91 | 3.47E-08 | 2.79E-05 |
| cg20758953 | ZNF74 | 3'UTR | S_Shore | 0.88 | 0.90 | 3.50E-08 | 2.81E-05 |
| cg18205465 | C7orf40 | TSS1500 | Island | 0.07 | 0.11 | 3.52E-08 | 2.82E-05 |
| cg02618319 | USP2 | 5'UTR |  | 0.96 | 0.95 | 3.53E-08 | 2.83E-05 |
| cg18075287 | GABARAPL1 | TSS200 | Island | 0.27 | 0.25 | 3.55E-08 | 2.84E-05 |
| cg22403782 | ALPP | Body | Island | 0.31 | 0.37 | 3.56E-08 | 2.84E-05 |
| cg18766608 | DAGLA | Body | Island | 0.89 | 0.91 | 3.57E-08 | 2.84E-05 |
| cg07153264 | FBXL19 | Body | S_Shore | 0.97 | 0.96 | 3.60E-08 | 2.86E-05 |
| cg04908325 | ZNF410 | TSS200 | Island | 0.05 | 0.04 | 3.61E-08 | 2.86E-05 |
| cg18118795 | RBBP4 | Body | S_Shore | 0.47 | 0.53 | 3.62E-08 | 2.86E-05 |
| cg14312334 | CIT | Body | Island | 0.96 | 0.95 | 3.62E-08 | 2.86E-05 |
| cg13324539 | |  |  | 0.76 | 0.73 | 3.63E-08 | 2.87E-05 |
| cg05985501 | |  | Island | 0.65 | 0.67 | 3.63E-08 | 2.87E-05 |
| cg18499636 | ARHGEF10 | Body | S_Shore | 0.82 | 0.84 | 3.70E-08 | 2.91E-05 |
| cg08924554 | IGFBP7 | TSS1500 | Island | 0.07 | 0.10 | 3.73E-08 | 2.93E-05 |
| cg09299076 | ZNF276 | Body |  | 0.93 | 0.91 | 3.74E-08 | 2.93E-05 |
| cg07855322 | |  |  | 0.76 | 0.73 | 3.75E-08 | 2.94E-05 |
| cg10326891 | NUDT9 | 1stExon | Island | 0.12 | 0.15 | 3.75E-08 | 2.94E-05 |
| cg06361278 | |  | Island | 0.29 | 0.34 | 3.89E-08 | 3.04E-05 |
| cg06658468 | OTOP1 | TSS1500 | Island | 0.22 | 0.28 | 3.93E-08 | 3.06E-05 |
| cg06616029 | TTBK2 | TSS200 | Island | 0.05 | 0.04 | 3.95E-08 | 3.08E-05 |
| cg07804408 | |  |  | 0.86 | 0.81 | 3.96E-08 | 3.08E-05 |
| cg07104380 | |  |  | 0.73 | 0.71 | 3.98E-08 | 3.09E-05 |
| cg07495755 | |  | Island | 0.68 | 0.67 | 4.00E-08 | 3.11E-05 |
| cg14487577 | MIR133B | TSS1500 |  | 0.81 | 0.85 | 4.06E-08 | 3.14E-05 |
| cg20445034 | KCNH4 | Body |  | 0.97 | 0.96 | 4.06E-08 | 3.14E-05 |
| cg14606129 | |  |  | 0.71 | 0.78 | 4.07E-08 | 3.14E-05 |
| cg05066621 | RBP3 | 3'UTR |  | 0.71 | 0.75 | 4.09E-08 | 3.15E-05 |
| cg01419670 | |  | N_Shore | 0.51 | 0.57 | 4.11E-08 | 3.16E-05 |
| cg14681176 | SIRT7 | Body | N_Shelf | 0.97 | 0.96 | 4.13E-08 | 3.17E-05 |
| cg19165344 | AP1B1 | TSS1500 | S_Shore | 0.73 | 0.69 | 4.15E-08 | 3.18E-05 |
| cg11648740 | FLJ35220 | 3'UTR |  | 0.77 | 0.81 | 4.18E-08 | 3.19E-05 |
| cg00887547 | TAOK3 | TSS1500 | Island | 0.09 | 0.10 | 4.18E-08 | 3.19E-05 |
| cg13642800 | |  |  | 0.69 | 0.67 | 4.19E-08 | 3.20E-05 |
| cg24865495 | MACROD1 | Body |  | 0.96 | 0.95 | 4.21E-08 | 3.20E-05 |
| cg18206858 | |  |  | 0.86 | 0.88 | 4.21E-08 | 3.20E-05 |
| cg07141622 | |  | Island | 0.14 | 0.12 | 4.26E-08 | 3.23E-05 |
| cg23463205 | PNLIP | 5'UTR |  | 0.83 | 0.76 | 4.26E-08 | 3.23E-05 |
| cg05192538 | |  | Island | 0.85 | 0.87 | 4.26E-08 | 3.23E-05 |
| cg19134728 | JAKMIP3 | Body | N_Shore | 0.81 | 0.84 | 4.29E-08 | 3.24E-05 |
| cg20665002 | ATP8A2 | 1stExon | Island | 0.12 | 0.11 | 4.34E-08 | 3.28E-05 |
| cg06866416 | LOC595101 | TSS200 | Island | 0.05 | 0.04 | 4.36E-08 | 3.28E-05 |
| cg09934692 | FIGNL2 | 1stExon | N_Shelf | 0.85 | 0.87 | 4.36E-08 | 3.28E-05 |
| cg17448109 | RNF115 | Body |  | 0.92 | 0.90 | 4.38E-08 | 3.28E-05 |
| cg23170535 | |  | S_Shelf | 0.84 | 0.86 | 4.38E-08 | 3.28E-05 |
| cg12661610 | RGS12 | Body | N_Shelf | 0.69 | 0.72 | 4.38E-08 | 3.28E-05 |
| cg02423267 | |  |  | 0.89 | 0.87 | 4.40E-08 | 3.29E-05 |
| cg13532410 | CACNA2D3 | Body |  | 0.61 | 0.68 | 4.43E-08 | 3.31E-05 |
| cg23208326 | MTHFD2L | Body |  | 0.78 | 0.83 | 4.45E-08 | 3.32E-05 |
| cg23243012 | PROKR1 | Body | Island | 0.94 | 0.93 | 4.51E-08 | 3.36E-05 |
| cg04400496 | |  |  | 0.74 | 0.67 | 4.58E-08 | 3.40E-05 |
| cg10542127 | |  | S_Shelf | 0.42 | 0.46 | 4.60E-08 | 3.42E-05 |
| cg21889116 | TMEM68 | 5'UTR | Island | 0.10 | 0.08 | 4.64E-08 | 3.44E-05 |
| cg19435453 | |  |  | 0.95 | 0.92 | 4.67E-08 | 3.45E-05 |
| cg01739965 | VPS54 | TSS200 | Island | 0.10 | 0.12 | 4.67E-08 | 3.45E-05 |
| cg27551895 | |  | S_Shore | 0.14 | 0.13 | 4.69E-08 | 3.46E-05 |
| cg02340915 | KDM2B | Body | N_Shore | 0.08 | 0.10 | 4.71E-08 | 3.47E-05 |
| cg14412794 | WWC2 | Body |  | 0.87 | 0.90 | 4.72E-08 | 3.47E-05 |
| cg13424330 | HAUS8 | TSS200 | Island | 0.13 | 0.10 | 4.74E-08 | 3.48E-05 |
| cg04951822 | OAS1 | Body |  | 0.33 | 0.41 | 4.76E-08 | 3.48E-05 |
| cg12219123 | AKR7A2 | 1stExon | Island | 0.02 | 0.03 | 4.76E-08 | 3.48E-05 |
| cg14153876 | GPD2 | 5'UTR | S_Shore | 0.13 | 0.11 | 4.76E-08 | 3.48E-05 |
| cg20330023 | ARNT2 | Body |  | 0.77 | 0.80 | 4.76E-08 | 3.48E-05 |
| cg14860917 | PSD3 | TSS1500 | Island | 0.42 | 0.46 | 4.79E-08 | 3.49E-05 |
| cg09725686 | |  | S_Shelf | 0.84 | 0.86 | 4.81E-08 | 3.50E-05 |
| cg23348743 | EBF1 | Body |  | 0.72 | 0.77 | 4.82E-08 | 3.50E-05 |
| cg00068377 | PRDM16 | Body | Island | 0.98 | 0.97 | 4.83E-08 | 3.50E-05 |
| cg18748085 | CDH12 | 5'UTR |  | 0.78 | 0.82 | 4.84E-08 | 3.50E-05 |
| cg09794469 | TNRC18 | Body | Island | 0.95 | 0.91 | 4.84E-08 | 3.50E-05 |
| cg15369419 | KLF7 | 3'UTR |  | 0.87 | 0.82 | 4.87E-08 | 3.52E-05 |
| cg18311516 | DYNC1H1 | Body |  | 0.96 | 0.95 | 4.88E-08 | 3.52E-05 |
| cg11930700 | |  |  | 0.75 | 0.70 | 4.88E-08 | 3.52E-05 |
| cg27130993 | ABLIM3 | Body |  | 0.54 | 0.62 | 4.91E-08 | 3.53E-05 |
| cg01922433 | ADAMTSL4 | Body | Island | 0.93 | 0.92 | 4.92E-08 | 3.53E-05 |
| cg03708990 | OSBPL7 | TSS1500 |  | 0.73 | 0.76 | 4.94E-08 | 3.53E-05 |
| cg16157016 | PICK1 | Body | Island | 0.97 | 0.95 | 4.94E-08 | 3.53E-05 |
| cg20014778 | URB2 | TSS1500 | Island | 0.24 | 0.22 | 4.96E-08 | 3.54E-05 |
| cg16494843 | |  |  | 0.87 | 0.82 | 5.01E-08 | 3.57E-05 |
| cg25371449 | SNHG3-RCC1 | Body | Island | 0.94 | 0.93 | 5.05E-08 | 3.60E-05 |
| cg10887309 | PRKAG1 | TSS200 | Island | 0.21 | 0.18 | 5.09E-08 | 3.61E-05 |
| cg08431873 | C11orf73 | TSS1500 |  | 0.07 | 0.08 | 5.09E-08 | 3.61E-05 |
| cg19889666 | TP53AIP1 | TSS200 |  | 0.75 | 0.78 | 5.09E-08 | 3.61E-05 |
| cg07589519 | MACROD1 | Body | S_Shore | 0.82 | 0.84 | 5.10E-08 | 3.61E-05 |
| cg24379915 | DUSP4 | Body | N_Shelf | 0.77 | 0.81 | 5.14E-08 | 3.64E-05 |
| cg02933375 | RPTOR | Body | S_Shore | 0.96 | 0.94 | 5.24E-08 | 3.70E-05 |
| cg27182159 | RPS18 | Body | S_Shore | 0.50 | 0.57 | 5.24E-08 | 3.70E-05 |
| cg00794722 | |  |  | 0.61 | 0.58 | 5.30E-08 | 3.73E-05 |
| cg26900750 | LTBP4 | Body | Island | 0.17 | 0.15 | 5.31E-08 | 3.73E-05 |
| cg08141342 | PTCH2 | TSS1500 | Island | 0.10 | 0.08 | 5.32E-08 | 3.74E-05 |
| cg23117592 | UNC80 | Body | Island | 0.92 | 0.87 | 5.34E-08 | 3.74E-05 |
| cg08704623 | |  |  | 0.66 | 0.62 | 5.34E-08 | 3.74E-05 |
| cg10553894 | CPT1A | Body | Island | 0.95 | 0.93 | 5.42E-08 | 3.79E-05 |
| cg23817637 | CLRN3 | TSS1500 |  | 0.55 | 0.66 | 5.44E-08 | 3.80E-05 |
| cg11925103 | NDUFS7 | Body | Island | 0.98 | 0.97 | 5.45E-08 | 3.80E-05 |
| cg04822973 | ARG2 | TSS1500 | Island | 0.13 | 0.11 | 5.47E-08 | 3.81E-05 |
| cg16385237 | NEU3 | 5'UTR | Island | 0.14 | 0.13 | 5.50E-08 | 3.82E-05 |
| cg07277624 | DBNDD2 | TSS200 | Island | 0.10 | 0.08 | 5.51E-08 | 3.82E-05 |
| cg18461093 | |  |  | 0.62 | 0.60 | 5.52E-08 | 3.83E-05 |
| cg23879460 | LOC285370 | TSS1500 |  | 0.70 | 0.74 | 5.56E-08 | 3.85E-05 |
| cg17998283 | |  | S_Shelf | 0.71 | 0.77 | 5.60E-08 | 3.87E-05 |
| cg21696055 | ARHGEF10L | Body |  | 0.80 | 0.82 | 5.60E-08 | 3.87E-05 |
| cg14007706 | SLC25A23 | Body | N_Shore | 0.60 | 0.64 | 5.68E-08 | 3.92E-05 |
| cg15464363 | DEPDC5 | TSS1500 | Island | 0.27 | 0.24 | 5.72E-08 | 3.93E-05 |
| cg27194586 | |  | Island | 0.15 | 0.13 | 5.74E-08 | 3.94E-05 |
| cg05093469 | FLVCR2 | Body |  | 0.94 | 0.92 | 5.75E-08 | 3.94E-05 |
| cg14414124 | PPP3R1 | Body |  | 0.40 | 0.45 | 5.76E-08 | 3.94E-05 |
| cg09331995 | |  |  | 0.59 | 0.53 | 5.77E-08 | 3.94E-05 |
| cg05179805 | |  |  | 0.19 | 0.17 | 5.77E-08 | 3.94E-05 |
| cg10778931 | SCAPER | Body |  | 0.83 | 0.80 | 5.79E-08 | 3.95E-05 |
| cg03915940 | C16orf90 | Body | N_Shore | 0.70 | 0.74 | 5.80E-08 | 3.95E-05 |
| cg21539981 | |  |  | 0.96 | 0.94 | 5.86E-08 | 3.98E-05 |
| cg08993103 | SEL1L3 | Body |  | 0.84 | 0.87 | 5.86E-08 | 3.98E-05 |
| cg26741280 | SLC6A4 | TSS200 | Island | 0.19 | 0.17 | 5.86E-08 | 3.98E-05 |
| cg18821320 | BAT1 | 1stExon | Island | 0.08 | 0.10 | 5.87E-08 | 3.98E-05 |
| cg04722977 | ZNF526 | TSS200 | Island | 0.12 | 0.10 | 5.88E-08 | 3.98E-05 |
| cg16278828 | MAN2C1 | Body | Island | 0.93 | 0.91 | 5.89E-08 | 3.98E-05 |
| cg13852730 | |  |  | 0.79 | 0.83 | 5.90E-08 | 3.98E-05 |
| cg19153095 | ZFPM2 | Body |  | 0.68 | 0.74 | 5.90E-08 | 3.98E-05 |
| cg23430209 | HCCA2 | Body |  | 0.97 | 0.96 | 5.92E-08 | 3.98E-05 |
| cg18824724 | SORBS2 | 5'UTR |  | 0.82 | 0.86 | 5.92E-08 | 3.98E-05 |
| cg06060868 | SDHA | Body |  | 0.73 | 0.77 | 5.96E-08 | 4.00E-05 |
| cg13849727 | C9orf93 | Body |  | 0.85 | 0.81 | 5.98E-08 | 4.01E-05 |
| cg04716530 | ITGAL | Body | Island | 0.69 | 0.76 | 6.00E-08 | 4.02E-05 |
| cg13709054 | DOM3Z | 5'UTR | Island | 0.24 | 0.22 | 6.02E-08 | 4.02E-05 |
| cg13502545 | |  |  | 0.83 | 0.86 | 6.06E-08 | 4.05E-05 |
| cg16261572 | CBY3 | TSS1500 | S_Shelf | 0.85 | 0.88 | 6.07E-08 | 4.05E-05 |
| cg14430629 | RNF112 | TSS1500 |  | 0.77 | 0.80 | 6.08E-08 | 4.05E-05 |
| cg23313266 | |  |  | 0.69 | 0.73 | 6.10E-08 | 4.05E-05 |
| cg08371086 | FAM19A1 | Body |  | 0.85 | 0.82 | 6.11E-08 | 4.06E-05 |
| cg01785490 | |  |  | 0.72 | 0.77 | 6.15E-08 | 4.08E-05 |
| cg22123804 | |  | Island | 0.76 | 0.69 | 6.16E-08 | 4.08E-05 |
| cg01233786 | ATP11A | Body | S_Shore | 0.98 | 0.98 | 6.18E-08 | 4.09E-05 |
| cg12384262 | PFKL | Body | Island | 0.97 | 0.96 | 6.19E-08 | 4.09E-05 |
| cg01592662 | NTAN1 | TSS1500 | S_Shore | 0.07 | 0.09 | 6.21E-08 | 4.09E-05 |
| cg12752325 | |  |  | 0.60 | 0.65 | 6.22E-08 | 4.09E-05 |
| cg10446401 | RPL31 | Body | S_Shore | 0.14 | 0.17 | 6.23E-08 | 4.09E-05 |
| cg03859106 | |  |  | 0.84 | 0.80 | 6.23E-08 | 4.09E-05 |
| cg12623107 | GTF2H1 | 5'UTR | S_Shelf | 0.82 | 0.87 | 6.27E-08 | 4.11E-05 |
| cg25295726 | FUT11 | TSS200 | Island | 0.14 | 0.13 | 6.30E-08 | 4.12E-05 |
| cg14614754 | |  |  | 0.63 | 0.71 | 6.31E-08 | 4.12E-05 |
| cg16677528 | IL1F7 | Body |  | 0.82 | 0.84 | 6.31E-08 | 4.12E-05 |
| cg04337618 | HAGHL | TSS200 | Island | 0.23 | 0.30 | 6.32E-08 | 4.12E-05 |
| cg24385334 | ARHGAP22 | Body | Island | 0.04 | 0.02 | 6.35E-08 | 4.14E-05 |
| cg00959749 | TMEM48 | Body | N_Shore | 0.66 | 0.72 | 6.38E-08 | 4.15E-05 |
| cg24340655 | |  |  | 0.78 | 0.82 | 6.40E-08 | 4.15E-05 |
| cg15911114 | FST | 3'UTR | S_Shelf | 0.72 | 0.79 | 6.41E-08 | 4.15E-05 |
| cg18910630 | TFCP2L1 | Body | N_Shelf | 0.72 | 0.76 | 6.42E-08 | 4.15E-05 |
| cg11543196 | CCDC49 | Body | S_Shelf | 0.90 | 0.91 | 6.42E-08 | 4.15E-05 |
| cg08524717 | |  | N_Shore | 0.76 | 0.70 | 6.42E-08 | 4.15E-05 |
| cg08099570 | CNGB1 | Body | Island | 0.62 | 0.65 | 6.44E-08 | 4.16E-05 |
| cg17842912 | CXXC5 | Body | Island | 0.96 | 0.94 | 6.51E-08 | 4.20E-05 |
| cg08240592 | C1orf107 | 3'UTR |  | 0.76 | 0.78 | 6.54E-08 | 4.20E-05 |
| cg00086809 | RAB40C | 1stExon | Island | 0.09 | 0.11 | 6.54E-08 | 4.20E-05 |
| cg15386853 | PRDM16 | Body | N_Shore | 0.73 | 0.76 | 6.55E-08 | 4.20E-05 |
| cg09801837 | |  | N_Shore | 0.81 | 0.85 | 6.57E-08 | 4.21E-05 |
| cg06026375 | PMCH | 1stExon |  | 0.79 | 0.83 | 6.60E-08 | 4.22E-05 |
| cg03621406 | FBXL12 | TSS1500 | Island | 0.12 | 0.15 | 6.60E-08 | 4.22E-05 |
| cg15734706 | RARG | 1stExon | Island | 0.08 | 0.06 | 6.60E-08 | 4.22E-05 |
| cg25618672 | |  | Island | 0.96 | 0.94 | 6.62E-08 | 4.22E-05 |
| cg27536870 | |  |  | 0.76 | 0.79 | 6.63E-08 | 4.22E-05 |
| cg01700035 | ZNF668 | 5'UTR | S_Shelf | 0.49 | 0.45 | 6.65E-08 | 4.22E-05 |
| cg08062329 | PRR3 | 5'UTR | Island | 0.05 | 0.05 | 6.65E-08 | 4.22E-05 |
| cg25490241 | |  |  | 0.87 | 0.90 | 6.65E-08 | 4.22E-05 |
| cg11814935 | RABIF | TSS200 | Island | 0.05 | 0.03 | 6.66E-08 | 4.22E-05 |
| cg00475815 | SENP5 | 1stExon | Island | 0.14 | 0.12 | 6.71E-08 | 4.25E-05 |
| cg22153062 | TUBB6 | Body | N_Shore | 0.46 | 0.42 | 6.74E-08 | 4.26E-05 |
| cg01209150 | |  |  | 0.79 | 0.83 | 6.77E-08 | 4.27E-05 |
| cg26346930 | |  |  | 0.85 | 0.87 | 6.78E-08 | 4.27E-05 |
| cg14429979 | AP2A2 | Body | N_Shore | 0.84 | 0.87 | 6.79E-08 | 4.27E-05 |
| cg23252848 | YBX1 | TSS1500 | Island | 0.06 | 0.05 | 6.82E-08 | 4.29E-05 |
| cg13286990 | LOC100132831 | TSS1500 | S_Shore | 0.77 | 0.81 | 6.86E-08 | 4.31E-05 |
| cg20152152 | GPR152 | 1stExon |  | 0.96 | 0.95 | 6.96E-08 | 4.37E-05 |
| cg23555340 | |  | Island | 0.19 | 0.17 | 7.01E-08 | 4.39E-05 |
| cg12482260 | NDUFAF4 | TSS200 | Island | 0.06 | 0.05 | 7.05E-08 | 4.41E-05 |
| cg10716494 | MAML2 | 5'UTR |  | 0.17 | 0.15 | 7.11E-08 | 4.44E-05 |
| cg12279175 | |  |  | 0.79 | 0.83 | 7.15E-08 | 4.46E-05 |
| cg24876404 | |  |  | 0.59 | 0.65 | 7.16E-08 | 4.46E-05 |
| cg12876356 | GFI1 | Body | Island | 0.75 | 0.84 | 7.19E-08 | 4.47E-05 |
| cg14878128 | ABCB5 | TSS200 |  | 0.70 | 0.77 | 7.25E-08 | 4.51E-05 |
| cg05331472 | BCAS4 | Body | N_Shore | 0.78 | 0.81 | 7.28E-08 | 4.51E-05 |
| cg06991955 | TSC2 | Body | Island | 0.95 | 0.93 | 7.28E-08 | 4.51E-05 |
| cg02759193 | |  | N_Shore | 0.79 | 0.83 | 7.29E-08 | 4.51E-05 |
| cg25147684 | GPR78 | Body | Island | 0.74 | 0.77 | 7.29E-08 | 4.51E-05 |
| cg26177629 | RAB18 | Body | Island | 0.12 | 0.16 | 7.31E-08 | 4.51E-05 |
| cg07399636 | |  |  | 0.82 | 0.86 | 7.33E-08 | 4.52E-05 |
| cg02832305 | FCGR2C | Body |  | 0.93 | 0.91 | 7.33E-08 | 4.52E-05 |
| cg07267166 | ZNF323 | 1stExon |  | 0.10 | 0.15 | 7.35E-08 | 4.52E-05 |
| cg06989074 | LRP5 | Body |  | 0.91 | 0.88 | 7.37E-08 | 4.52E-05 |
| cg07346171 | EPB41 | 5'UTR |  | 0.85 | 0.89 | 7.37E-08 | 4.52E-05 |
| cg19834585 | GALNT9 | Body | Island | 0.70 | 0.74 | 7.41E-08 | 4.53E-05 |
| cg18831262 | PNPLA6 | TSS1500 | Island | 0.11 | 0.09 | 7.41E-08 | 4.53E-05 |
| cg27118937 | LMF1 | Body | S_Shelf | 0.96 | 0.95 | 7.42E-08 | 4.53E-05 |
| cg23008083 | ITGB6 | TSS1500 |  | 0.84 | 0.86 | 7.42E-08 | 4.53E-05 |
| cg06280512 | PRKD2 | Body | Island | 0.05 | 0.07 | 7.44E-08 | 4.53E-05 |
| cg14781190 | SERINC2 | TSS200 | Island | 0.12 | 0.09 | 7.44E-08 | 4.53E-05 |
| cg25680105 | |  | Island | 0.72 | 0.62 | 7.46E-08 | 4.53E-05 |
| cg21223803 | DACT2 | Body | S_Shore | 0.72 | 0.75 | 7.47E-08 | 4.54E-05 |
| cg24412117 | LOC100188947 | Body | Island | 0.11 | 0.13 | 7.49E-08 | 4.54E-05 |
| cg08598287 | |  | N_Shore | 0.77 | 0.80 | 7.52E-08 | 4.56E-05 |
| cg10795359 | AKR1B1 | TSS200 | Island | 0.20 | 0.19 | 7.57E-08 | 4.58E-05 |
| cg02706575 | CCL13 | TSS1500 |  | 0.81 | 0.84 | 7.63E-08 | 4.61E-05 |
| cg17306814 | SLC7A14 | 5'UTR |  | 0.83 | 0.86 | 7.64E-08 | 4.61E-05 |
| cg16681031 | RNF213 | Body | S_Shore | 0.97 | 0.96 | 7.67E-08 | 4.62E-05 |
| cg20536794 | |  |  | 0.80 | 0.83 | 7.73E-08 | 4.65E-05 |
| cg10094238 | ARHGAP27 | 1stExon | Island | 0.92 | 0.91 | 7.76E-08 | 4.66E-05 |
| cg12168066 | |  |  | 0.81 | 0.85 | 7.77E-08 | 4.66E-05 |
| cg08740477 | FER | 5'UTR | Island | 0.06 | 0.05 | 7.77E-08 | 4.66E-05 |
| cg25673945 | |  |  | 0.60 | 0.58 | 7.79E-08 | 4.66E-05 |
| cg06500120 | GSX2 | 1stExon | Island | 0.07 | 0.06 | 7.80E-08 | 4.66E-05 |
| cg25987208 | |  | S_Shore | 0.34 | 0.28 | 7.80E-08 | 4.66E-05 |
| cg01769354 | |  |  | 0.55 | 0.58 | 7.84E-08 | 4.68E-05 |
| cg24162965 | |  |  | 0.94 | 0.92 | 7.87E-08 | 4.69E-05 |
| cg09048334 | |  | Island | 0.16 | 0.13 | 7.88E-08 | 4.69E-05 |
| cg23963476 | SMARCA4 | Body | S_Shore | 0.83 | 0.85 | 7.91E-08 | 4.70E-05 |
| cg17750043 | TMEM211 | TSS200 |  | 0.86 | 0.88 | 7.91E-08 | 4.70E-05 |
| cg20752695 | |  |  | 0.65 | 0.69 | 7.95E-08 | 4.72E-05 |
| cg22857604 | RASSF5 | Body | Island | 0.12 | 0.10 | 7.97E-08 | 4.72E-05 |
| cg27063327 | BCAS3 | Body |  | 0.86 | 0.88 | 7.99E-08 | 4.72E-05 |
| cg11023992 | OR7E156P | TSS200 |  | 0.83 | 0.86 | 7.99E-08 | 4.72E-05 |
| cg12747410 | MS4A15 | TSS1500 |  | 0.82 | 0.85 | 7.99E-08 | 4.72E-05 |
| cg00681665 | ALOX12B | TSS1500 |  | 0.71 | 0.65 | 8.01E-08 | 4.72E-05 |
| cg17287155 | AHRR | Body |  | 0.76 | 0.81 | 8.06E-08 | 4.74E-05 |
| cg00918738 | TCF15 | Body | N_Shore | 0.79 | 0.82 | 8.06E-08 | 4.74E-05 |
| cg11130097 | C10orf75 | Body | S_Shelf | 0.69 | 0.66 | 8.06E-08 | 4.74E-05 |
| cg24408603 | TOR1AIP1 | TSS200 | N_Shore | 0.07 | 0.06 | 8.07E-08 | 4.74E-05 |
| cg03299095 | SNORD115-11 | TSS200 |  | 0.96 | 0.95 | 8.13E-08 | 4.76E-05 |
| cg17727418 | NRG3 | Body |  | 0.72 | 0.68 | 8.14E-08 | 4.76E-05 |
| cg05575273 | |  |  | 0.95 | 0.93 | 8.14E-08 | 4.76E-05 |
| cg15009352 | HSPG2 | TSS200 |  | 0.12 | 0.11 | 8.15E-08 | 4.76E-05 |
| cg05213267 | DIP2C | Body | N_Shore | 0.92 | 0.90 | 8.17E-08 | 4.76E-05 |
| cg14910395 | GRIN2D | Body | N_Shelf | 0.70 | 0.73 | 8.18E-08 | 4.77E-05 |
| cg13444392 | |  |  | 0.87 | 0.90 | 8.20E-08 | 4.77E-05 |
| cg05107228 | HDAC4 | Body |  | 0.98 | 0.98 | 8.21E-08 | 4.77E-05 |
| cg24732062 | |  |  | 0.78 | 0.76 | 8.23E-08 | 4.78E-05 |
| cg05844366 | |  |  | 0.72 | 0.64 | 8.25E-08 | 4.78E-05 |
| cg14787287 | ABCA4 | TSS1500 |  | 0.89 | 0.91 | 8.27E-08 | 4.79E-05 |
| cg12617684 | |  |  | 0.67 | 0.72 | 8.32E-08 | 4.81E-05 |
| cg25747655 | |  |  | 0.71 | 0.75 | 8.33E-08 | 4.81E-05 |
| cg25766801 | CSMD1 | Body |  | 0.78 | 0.81 | 8.33E-08 | 4.81E-05 |
| cg21734175 | |  |  | 0.52 | 0.58 | 8.35E-08 | 4.81E-05 |
| cg07604202 | ZFP64 | Body | Island | 0.72 | 0.67 | 8.42E-08 | 4.85E-05 |
| cg09749862 | LRP1 | Body |  | 0.98 | 0.97 | 8.47E-08 | 4.87E-05 |
| cg24926253 | SLC22A16 | TSS1500 | S_Shore | 0.82 | 0.77 | 8.51E-08 | 4.89E-05 |
| cg20297940 | |  |  | 0.82 | 0.80 | 8.52E-08 | 4.89E-05 |
| cg08079331 | TMCC3 | Body |  | 0.86 | 0.88 | 8.57E-08 | 4.91E-05 |
| cg01476047 | ASCC1 | 5'UTR | N_Shore | 0.80 | 0.85 | 8.61E-08 | 4.92E-05 |
| cg12660445 | SNORD18A | TSS200 |  | 0.46 | 0.51 | 8.61E-08 | 4.92E-05 |
| cg16555896 | MORN1 | Body | Island | 0.81 | 0.84 | 8.61E-08 | 4.92E-05 |
| cg10415122 | CSPG4 | Body | Island | 0.77 | 0.71 | 8.70E-08 | 4.96E-05 |
| cg24790419 | KIAA1683 | TSS1500 |  | 0.75 | 0.78 | 8.76E-08 | 4.99E-05 |
| cg26923014 | |  |  | 0.72 | 0.68 | 8.77E-08 | 4.99E-05 |
| cg02423618 | SPATA8 | TSS1500 |  | 0.57 | 0.61 | 8.80E-08 | 5.00E-05 |
| cg01552711 | IL17D | Body |  | 0.83 | 0.85 | 8.84E-08 | 5.02E-05 |
| cg16280946 | |  | Island | 0.68 | 0.64 | 8.86E-08 | 5.02E-05 |
| cg17872658 | RPTOR | Body | N_Shore | 0.83 | 0.86 | 8.88E-08 | 5.02E-05 |
| cg03930929 | LRRN1 | 5'UTR |  | 0.65 | 0.73 | 8.89E-08 | 5.02E-05 |
| cg02709840 | CBFB | TSS200 | Island | 0.13 | 0.17 | 8.89E-08 | 5.02E-05 |
| cg15034413 | |  |  | 0.85 | 0.87 | 8.90E-08 | 5.02E-05 |
| cg03485672 | |  |  | 0.86 | 0.88 | 8.92E-08 | 5.03E-05 |
| cg08773029 | ANKMY1 | Body |  | 0.96 | 0.95 | 8.95E-08 | 5.04E-05 |
| cg16724696 | HINFP | 5'UTR | Island | 0.12 | 0.11 | 9.07E-08 | 5.10E-05 |
| cg05880945 | C9orf122 | Body | Island | 0.21 | 0.25 | 9.10E-08 | 5.11E-05 |
| cg17820989 | MFSD3 | TSS200 | Island | 0.11 | 0.09 | 9.14E-08 | 5.13E-05 |
| cg05633605 | ANKRD55 | TSS1500 |  | 0.71 | 0.77 | 9.16E-08 | 5.13E-05 |
| cg05725703 | MYL3 | TSS1500 |  | 0.64 | 0.68 | 9.18E-08 | 5.13E-05 |
| cg25428612 | STAT4 | TSS1500 |  | 0.71 | 0.74 | 9.18E-08 | 5.13E-05 |
| cg07205462 | SNRPB2 | TSS200 | Island | 0.05 | 0.04 | 9.19E-08 | 5.13E-05 |
| cg09538725 | |  |  | 0.79 | 0.77 | 9.23E-08 | 5.14E-05 |
| cg19418648 | RGS14 | Body | Island | 0.61 | 0.65 | 9.24E-08 | 5.14E-05 |
| cg02283353 | POLRMT | Body | Island | 0.97 | 0.96 | 9.24E-08 | 5.14E-05 |
| cg01337429 | NTN3 | 1stExon | Island | 0.94 | 0.93 | 9.27E-08 | 5.16E-05 |
| cg03516394 | |  |  | 0.79 | 0.76 | 9.31E-08 | 5.17E-05 |
| cg15587018 | SUPT3H | Body |  | 0.77 | 0.75 | 9.31E-08 | 5.17E-05 |
| cg01704976 | TWF2 | TSS200 | Island | 0.04 | 0.02 | 9.34E-08 | 5.18E-05 |
| cg16834212 | SUCLG2 | TSS1500 | S_Shore | 0.30 | 0.35 | 9.35E-08 | 5.18E-05 |
| cg02047547 | ITPRIPL2 | 1stExon | Island | 0.87 | 0.83 | 9.37E-08 | 5.18E-05 |
| cg16754643 | ZNF605 | Body |  | 0.82 | 0.75 | 9.41E-08 | 5.20E-05 |
| cg27074221 | MIR495 | TSS200 |  | 0.72 | 0.78 | 9.42E-08 | 5.20E-05 |
| cg23473088 | |  |  | 0.81 | 0.84 | 9.43E-08 | 5.20E-05 |
| cg09740598 | SLC13A2 | Body |  | 0.83 | 0.85 | 9.46E-08 | 5.20E-05 |
| cg09075525 | |  |  | 0.78 | 0.73 | 9.47E-08 | 5.20E-05 |
| cg03329539 | |  | N_Shore | 0.33 | 0.38 | 9.48E-08 | 5.21E-05 |
| cg09376008 | |  |  | 0.19 | 0.24 | 9.52E-08 | 5.22E-05 |
| cg03793872 | |  | S_Shelf | 0.75 | 0.79 | 9.59E-08 | 5.25E-05 |
| cg15874144 | |  |  | 0.78 | 0.76 | 9.60E-08 | 5.25E-05 |
| cg01427976 | NDRG3 | TSS200 | Island | 0.05 | 0.04 | 9.61E-08 | 5.25E-05 |
| cg23516342 | DCAF8L2 | TSS1500 |  | 0.63 | 0.71 | 9.61E-08 | 5.25E-05 |
| cg03925157 | DSTN | TSS1500 | Island | 0.17 | 0.15 | 9.66E-08 | 5.27E-05 |
| cg08040148 | |  | S_Shelf | 0.62 | 0.56 | 9.72E-08 | 5.29E-05 |
| cg05853039 | MSI2 | Body | S_Shore | 0.18 | 0.25 | 9.75E-08 | 5.29E-05 |
| cg09834444 | SPIRE2 | 3'UTR | N_Shore | 0.58 | 0.56 | 9.75E-08 | 5.29E-05 |
| cg25468907 | TNNI1 | Body |  | 0.80 | 0.82 | 9.76E-08 | 5.29E-05 |
| cg02812891 | ECEL1P2 | TSS200 | Island | 0.61 | 0.57 | 9.76E-08 | 5.29E-05 |
| cg17628700 | MDK | TSS200 | N_Shore | 0.30 | 0.28 | 9.77E-08 | 5.29E-05 |
| cg27640712 | |  |  | 0.65 | 0.58 | 9.83E-08 | 5.32E-05 |
| cg10336578 | |  |  | 0.72 | 0.68 | 9.99E-08 | 5.40E-05 |
| cg03449125 | CAPN5 | 5'UTR | S_Shore | 0.82 | 0.84 | 1.00E-07 | 5.40E-05 |
| cg12720459 | PELI2 | 1stExon | Island | 0.15 | 0.18 | 1.00E-07 | 5.40E-05 |
| cg25265930 | HLA-DMB | Body |  | 0.38 | 0.33 | 1.00E-07 | 5.41E-05 |
| cg20986608 | SART1 | Body |  | 0.90 | 0.88 | 1.01E-07 | 5.41E-05 |
| cg01342858 | |  | Island | 0.84 | 0.81 | 1.01E-07 | 5.41E-05 |
| cg12806681 | AHRR | Body | N_Shore | 0.83 | 0.87 | 1.01E-07 | 5.42E-05 |
| cg14778576 | PPM1L | Body | S_Shelf | 0.89 | 0.90 | 1.01E-07 | 5.42E-05 |
| cg13471374 | RCL1 | Body | N_Shore | 0.97 | 0.95 | 1.01E-07 | 5.42E-05 |
| cg01123250 | UNC80 | Body | Island | 0.59 | 0.49 | 1.01E-07 | 5.42E-05 |
| cg15433604 | UPF1 | TSS1500 | Island | 0.04 | 0.02 | 1.02E-07 | 5.43E-05 |
| cg15110219 | PADI4 | 3'UTR |  | 0.80 | 0.83 | 1.02E-07 | 5.44E-05 |
| cg19998148 | CWF19L2 | TSS200 | Island | 0.05 | 0.04 | 1.03E-07 | 5.48E-05 |

All average methylation values are non-log transformed beta-values. Island status refers to the position of the probe relative to the island. Classes include: 1) Island, 2) N (north) shore, 3) S (south) shore, 4) N (north) shelf, 5) S (south) shelf and 6) blank denoting that the probe does not map to an island.
