# Supplementary material for: The effect of smoking on DNA methylation of peripheral blood mononuclear cells from African American women
Source: BMC Genomics. 2014 Feb 22;15:151. doi: 10.1186/1471-2164-15-151 (PMC3936875; doi:10.1186/1471-2164-15-151)
Supplement: Additional file 2 — Characteristics and average methylation levels at cg05575921 of a subset of 62 individuals used in an independent validation. [file 1471-2164-15-151-S2.docx]

Additional File 2. Table S1. Characteristics and average methylation levels from the Illumina platform and quantitative-PCR of the subset of female FACHS subjects used for the validation of differential methylation at cg05575921

|  | Smoker | Control |
| --- | --- | --- |
| Age | 48.9 ± 7 | 49.2 ± 10 |
| Smoking status | 29 | 33 |
| Average Illumina methylation | 0.68 ± 0.06 | 0.83 ± 0.02 |
| Average q-PCR methylation | 0.72 ± 0.11 | 1.00 ± 0.06 |
