# Supplementary material for: The effect of smoking on DNA methylation of peripheral blood mononuclear cells from African American women
Source: BMC Genomics. 2014 Feb 22;15:151. doi: 10.1186/1471-2164-15-151 (PMC3936875; doi:10.1186/1471-2164-15-151)
Supplement: Additional file 3: Figure S2 — Protein sub-networks identified by the miPALM algorithm and visualized in Cytoscape. [file 1471-2164-15-151-S3.pdf]

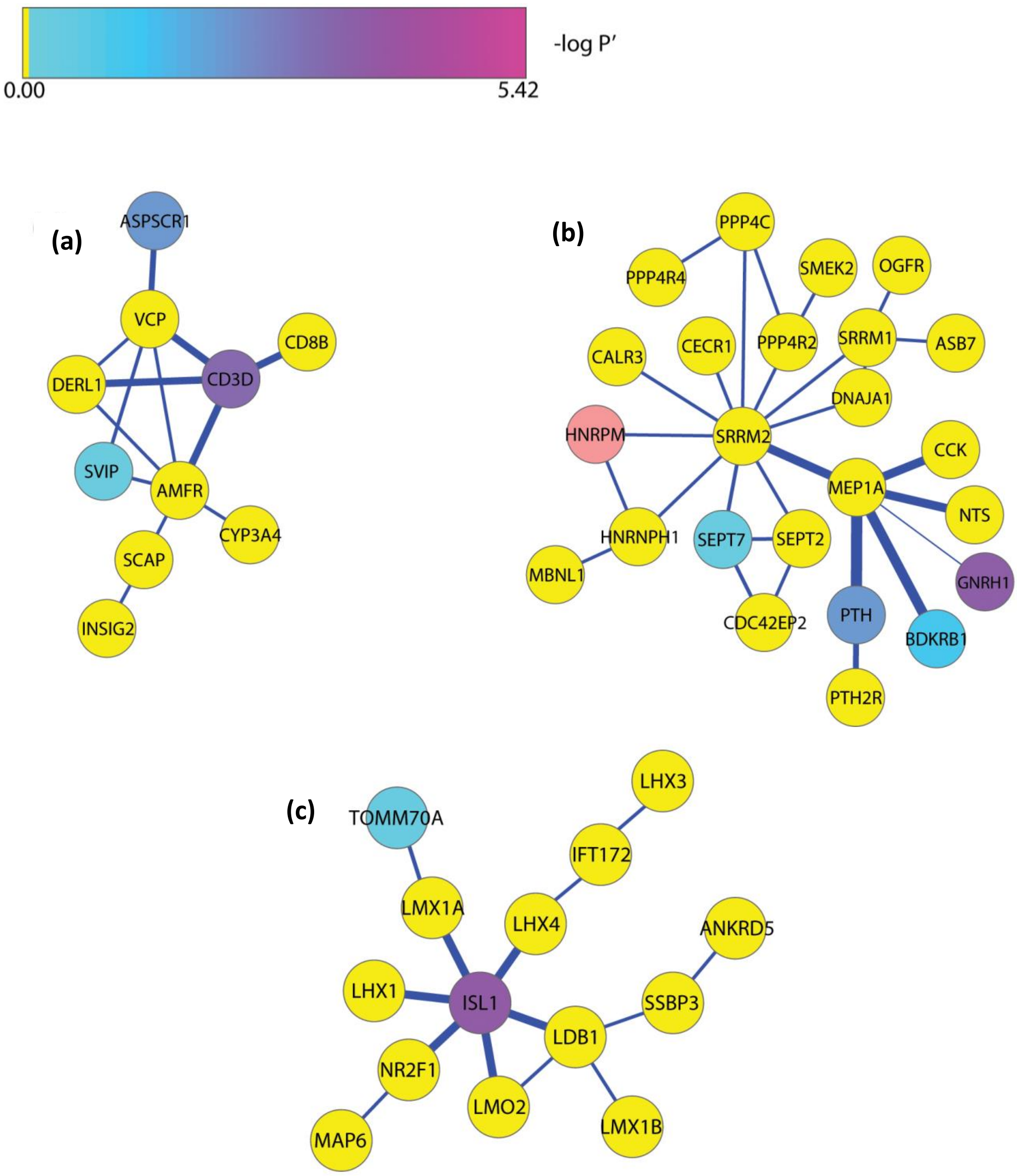

Additional File 3. Figure S1. Protein sub-networks identified by the miPALM algorithm and visualized using Cytoscape. Strength of the interaction between two proteins is depicted by line width. The color of each node represents the negative log p-value from the t-test (see color bar insert) between smokers and non-smokers after correction for multiple comparisons (-log P'). Sub-network **(a)** consists of 10 proteins ($p < 0.03$) enriched in cell signaling related pathway based on the BiNGO analysis. Sub-network **(b)** consists of 24 proteins ($p < 0.04$) enriched in RNA processing related pathway based on the BiNGO analysis. Sub-network **(c)** consists of 14 proteins ($p < 0.03$) enriched in central nervous system related pathway based on the BiNGO analysis.
