# Supplementary material for: The effect of smoking on DNA methylation of peripheral blood mononuclear cells from African American women
Source: BMC Genomics. 2014 Feb 22;15:151. doi: 10.1186/1471-2164-15-151 (PMC3936875; doi:10.1186/1471-2164-15-151)
Supplement: Additional file 5 — Gene ontology pathways of Additional file4: Figure S1(a) identified by the Cytoscape plugin BiNGO. [file 1471-2164-15-151-S5.docx]

Additional File 5. Table S3. Top 10 Pathways from BiNGO Pathway Analysis of Protein Sub-network depicted in Additional File 3 Figure S1(a)

Genes Corrected

GO Category Category Name Total Changed P-Value

GO:0006984 ER-nucleus signaling pathway 36 9 2.63E-9

GO:0034620 cellular response to unfolded protein 21 9 2.11E-5

GO:0030968 ER unfolded protein response 21 9 2.11E-5

GO:0071445 cellular response to protein stimulus 27 9 2.78E-5

GO:0030433 ER-associated protein catabolic process 27 9 2.78E-5

GO:0034976 response to endoplasmic reticulum 34 9 3.26E-5

GO:0071501 cellular response to sterol depletion 3 9 3.26E-5

GO:0006991 response to sterol depletion 3 9 3.26E-5

GO:0030967 ER-nuclear sterol response pathway 3 9 3.26E-5

GO:0071216 cellular response to biotic stimulus 49 9 8.69E-5
