# Supplementary material for: The effect of smoking on DNA methylation of peripheral blood mononuclear cells from African American women
Source: BMC Genomics. 2014 Feb 22;15:151. doi: 10.1186/1471-2164-15-151 (PMC3936875; doi:10.1186/1471-2164-15-151)
Supplement: Additional file 7 — Gene ontology pathways of Additional file4: Figure S1(c) identified by the Cytoscape plugin BiNGO. [file 1471-2164-15-151-S7.docx]

Additional File 7. Table S5. Top 10 Pathways from BiNGO Pathway Analysis of Protein Sub-network depicted in Additional File 3 Figure S1(c)

Genes Corrected

GO Category Category Name Total Changed P-Value

GO:0021953 CNS neuron differentiation 75 5 3.93E-7

GO:0021549 cerebellum development 30 4 6.47E-7

GO:0022037 metencephalon development 35 4 8.22E-7

GO:0030902 hindbrain development 58 4 4.95E-6

GO:0048513 organ development 1792 9 1.24E-5

GO:0021587 cerebellum morphogenesis 19 3 1.24E-5

GO:0007417 CNS development 443 6 1.24E-5

GO:0021575 hindbrain morphogenesis 23 3 1.87E-5

GO:0007275 multicellular organismal development 2972 10 3.70E-5

GO:0007420 brain development 303 5 4.32E-5
