# Supplementary material for: The effect of smoking on DNA methylation of peripheral blood mononuclear cells from African American women
Source: BMC Genomics. 2014 Feb 22;15:151. doi: 10.1186/1471-2164-15-151 (PMC3936875; doi:10.1186/1471-2164-15-151)
Supplement: Additional file 8 — Gene ontology pathways of Additional file3: Figure S2(a) identified by the Cytoscape plugin BiNGO. [file 1471-2164-15-151-S8.docx]

Additional File 8. Table S6. Top 10 Pathways from BiNGO Pathway Analysis of Protein Sub-network depicted in Additional File 4 Figure S2(a)

Genes Corrected

GO Category Category Name Total Changed P-Value

GO:0007041 lysosomal transport 27 3 7.60E-4

GO:0007034 vacuolar transport 31 3 7.60E-4

GO:0016192 vesicle-mediated transport 590 6 1.68E-3

GO:0008104 protein localization 921 7 1.68E-3

GO:0051234 establishment of localization 2611 10 3.25E-3

GO:0045022 early endosome to late endosome transport 11 2 3.25E-3

GO:0033036 macromolecule localization 1111 7 3.25E-3

GO:0045184 establishment of protein localization 767 6 3.54E-3

GO:0008333 endosome to lysosome transport 16 2 5.01E-3

GO:0007032 endosome organization 17 2 5.10E-3
