# Supplementary material for: The effect of smoking on DNA methylation of peripheral blood mononuclear cells from African American women
Source: BMC Genomics. 2014 Feb 22;15:151. doi: 10.1186/1471-2164-15-151 (PMC3936875; doi:10.1186/1471-2164-15-151)
Supplement: Additional file 9 — Gene ontology pathways of Additional file3: Figure S2(b) identified by the Cytoscape plugin BiNGO. [file 1471-2164-15-151-S9.docx]

Additional File 7. Table S5. Pathways from BiNGO Pathway Analysis of Protein Sub-network depicted in Additional File 2 Figure S2(b)

Genes Corrected

GO Category Category Name Total Changed P-Value

GO:0008380 RNA splicing 287 6 9.36E-4

GO:0006397 mRNA processing 309 6 9.36E-4

GO:0016071 mRNA metabolic process 369 6 1.73E-3

GO:0006396 RNA processing 568 6 1.46E-2

GO:0000375 RNA splicing, via transesterification reactions 99 3 3.66E-2
